# Two-sample Mendelian randomization investigating the causal association between endocarditis and osteomyelitis: A validation analysis using UK Biobank and FinnGen databases

**DOI:** 10.1097/MD.0000000000044651

**Published:** 2025-09-26

**Authors:** Huan Liu, Hao Xing, Xue Yu, Yanan Wang, Huancai Liu, Zhengqi Chang

**Affiliations:** a Department of Orthopedics, The 960th Hospital of PLA, Shandong Second Medical University, Jinan, Shandong, China; b School of Anesthesiology, Shandong Second Medical University, Weifang, Shandong, China; c Department of Joint Surgery, Affiliated Hospital of Shandong Second Medical University, School of Clinical Medicine, Shandong Second Medical University, Weifang, Shandong, China.

**Keywords:** infective endocarditis, Mendelian randomization, meta-analysis, osteomyelitis

## Abstract

We conducted this study to explore the causal relationship between infective endocarditis (IE) and osteomyelitis (OM). We conducted a systematic search of PubMed, Cochrane Library, Web of Science, and Embase for relevant studies up to January 10, 2025. A meta-analysis was performed to evaluate the co-occurrence rates of IE and OM. Subsequently, 2-sample Mendelian randomization (MR) and multivariable MR analyses were employed to explore potential causal relationships, with using GWAS data from the UK Biobank and FinnGen databases. Meta-analysis revealed that 14% of OM patients had concurrent IE (95% confidence intervals [CI] = 10–18%), while 6% of IE patients exhibited comorbid OM (95% CI = 4–9%). However, all MR analyses demonstrated non-significant causal associations between IE and OM. Pooled results from meta-analysis of inverse variance weighted estimates showed: when IE was the exposure and OM the outcome: odds ratio = 1.01 (95% CI = 0.96–1.07). When OM was the exposure and IE the outcome: odds ratio = 0.87 (95% CI = 0.75–1.00). Despite the statistically non-significant MR results, the clinically observed association between IE and OM cannot be dismissed. The mediating role of bacteremia in their relationship warrants further investigation.

## 1. Introduction

Infectious endocarditis (IE) and osteomyelitiss (OM) are 2 relatively rare and serious infectious diseases whose clinical association has long been of interest. IE is a disease defined as a condition of the endocardial surface of the heart in which the infection primarily involves heart valves (native or prosthetic), wall endocardium, ventricular septal defects, or indwelling cardiac devices.^[1]^ OM, on the other hand, has been described as a bone infection caused by direct spread of infection from adjacent tissues, direct injury to bone tissue due to surgery or trauma, and hematogenous dissemination triggered by systemic bacteremia.^[2,3]^ The main causative agent of both diseases is *Staphylococcus aureus*, and the incidence is increasing every year.^[4,5]^ In the United States, OM will occur in 21.8 cases per 100,000 people, and the incidence of OM will be higher in the elderly and diabetic patients.^[1,6]^ By 2019, the annual incidence of IE reached 13.8 cases per 100,000 people worldwide.^[3]^ Numerous clinical studies have observed that IE and OM occur concomitantly, suggesting a possible causal association. The guidelines^[7]^ state that clinicians should be highly suspicious of vertebral osteomyelitis (VOM) in the presence of IE, suggesting that IE may increase the risk of OM. However, the causal association between the 2 is not clear. This is because traditional observational studies have been unable to distinguish the direction of the association (e.g., whether IE causes OM or OM causes IE) and whether the association is driven by confounding factors (e.g., obesity and diabetes).

Mendelian randomization (MR) provides a novel approach to address this challenge. By utilizing genetic variants as instrumental variables (IV), MR can mimic randomized controlled trials, effectively circumventing confounding factors, and reverse causation. In recent years, the 2-sample MR design has further enabled the use of exposure and outcome data from independent cohorts, significantly enhancing research efficiency and feasibility. Additionally, multivariable MR helps mitigate confounding effects, thereby strengthening the reliability of findings. Current studies have demonstrated strong causal links between OM and CD^8+^ T cells^[8]^, type 2 diabetes^[9]^, human gut microbiota^[10]^, vitamin B6^[11]^, major depressive disorder,^[12]^ basal metabolic rate,^[13]^ and systemic lupus erythematosus,^[14]^ whereas no convincing causal relationship has been established between educational attainment^[15]^ and OM.

Both IE and OM are severe infectious diseases. Studies have reported that patients with concurrent conditions exhibit poor prognosis and elevated risk of treatment failure.^[16]^ Given the unclear causal relationship between IE and OM in current research, particularly the lack of MR studies, we conducted this investigation to explore their causal association. Furthermore, we performed a meta-analysis to examine the co-occurrence incidence risk of these conditions, aiming to enhance understanding of their epidemiological relationship. This study contributes novel insights into the complex interplay between OM and IE.

## 2. Methods

### 2.1. Meta-analysis

#### 2.1.1. Registration and reporting

While this investigation was conducted without prior protocol registration, methodological reporting rigorously adhered to the Preferred Reporting Items for Systematic Reviews and Meta-Analyses framework for transparent evidence synthesis.^[17]^

#### 2.1.2. Literature search

The search methodology encompassing 4 major biomedical databases (Medline via PubMed, Embase, Cochrane Library, and Web of Science) is comprehensively documented in Supplementary Material S1, Supplemental Digital Content, https://links.lww.com/MD/Q27. Our multi-platform query strategy covers indexed records up to January 10, 2025, combining controlled vocabulary descriptors, including MeSH terms such as “Osteomyelitis” [Mesh] and “Endocarditis” [Mesh], with relevant free-text terms to ensure comprehensive literature retrieval. To ensure comprehensive coverage of the literature, the references of the eligible articles were individually reviewed. Furthermore, post hoc inclusion criteria accommodated emerging evidence published during the manuscript preparation phase.

#### 2.1.3. Inclusion and exclusion criteria

We included studies investigating both OM and IE. Study designs encompassed both prospective and retrospective observational studies. The study populations comprised adults and pediatric patients. OM cases included both extremity OM and VOM. No language restrictions were applied during study selection. The prevalence of IE among OM patients is calculated by dividing the number of confirmed IE cases (n_endocarditis) by the total OM cohort (n_osteomyelitis) × 100%. The prevalence of OM among IE patients is calculated by dividing the number of confirmed OM cases (n_osteomyelitis) by the total IE cohort (n_endocarditis) × 100%.

Exclusion criteria comprised: studies with < 100 participants; exclusive focus on septic arthritis; unavailable outcome data; non-peer-reviewed materials (conference abstracts, protocols, letters), duplicate publications, and studies with inaccessible full-text articles.

#### 2.1.4. Data extraction

Two researchers conducted parallel literature screening and data extraction procedures. This workflow encompassed 3-stage evaluation (title → abstract → full text) of retrieved publications. Bibliographic records were processed and cataloged through EndNote v21 reference management system, with extracted information systematically archived in structured electronic spreadsheets for preliminary analysis. The selection protocol was implemented predetermined eligibility criteria. Extracted scholarly attributes underwent consistency validation, focusing on: primary investigator, publication year, geographical origin, research design, and participant cohort dimensions. The characteristics of the included studies are described in Supplementary Material S2, Supplemental Digital Content, https://links.lww.com/MD/Q27.

#### 2.1.5. Quality assessment

Included studies were evaluated for quality using the JBI Critical Appraisal Checklist for Case Series.^[18]^

#### 2.1.6. Statistical analysis

The meta-analysis was implemented in R 4.4.2 (Vienna, Austria; https://www.r-project.org/) using the meta package, synthesizing effect size with 95% confidence intervals (CI) through methodologically rigorous approaches. Proportional data underwent logit transformations for variance stabilization prior to pooling via the metaprop() function. Publication bias was evaluated through Egger regression asymmetry test in metabias() with 2-tailed α = 0.05, where *P* < .05 indicated statistically significant bias and *P* > .05 suggested minimal bias suspicion. Model selection adhered to heterogeneity thresholds quantified by *I*² statistics: fixed-effect models with inverse variance weighting (IVW) were applied when *I*² ≤ 50%, while random-effects models employing generalized linear mixed models were utilized for *I*² > 50%. Robustness was systematically verified through leave-one-out sensitivity analysis implemented in metainf(), which iteratively excluded individual studies and recalculated global estimates.

### 2.2. MR

#### 2.2.1. Study design

**Figure 1.**
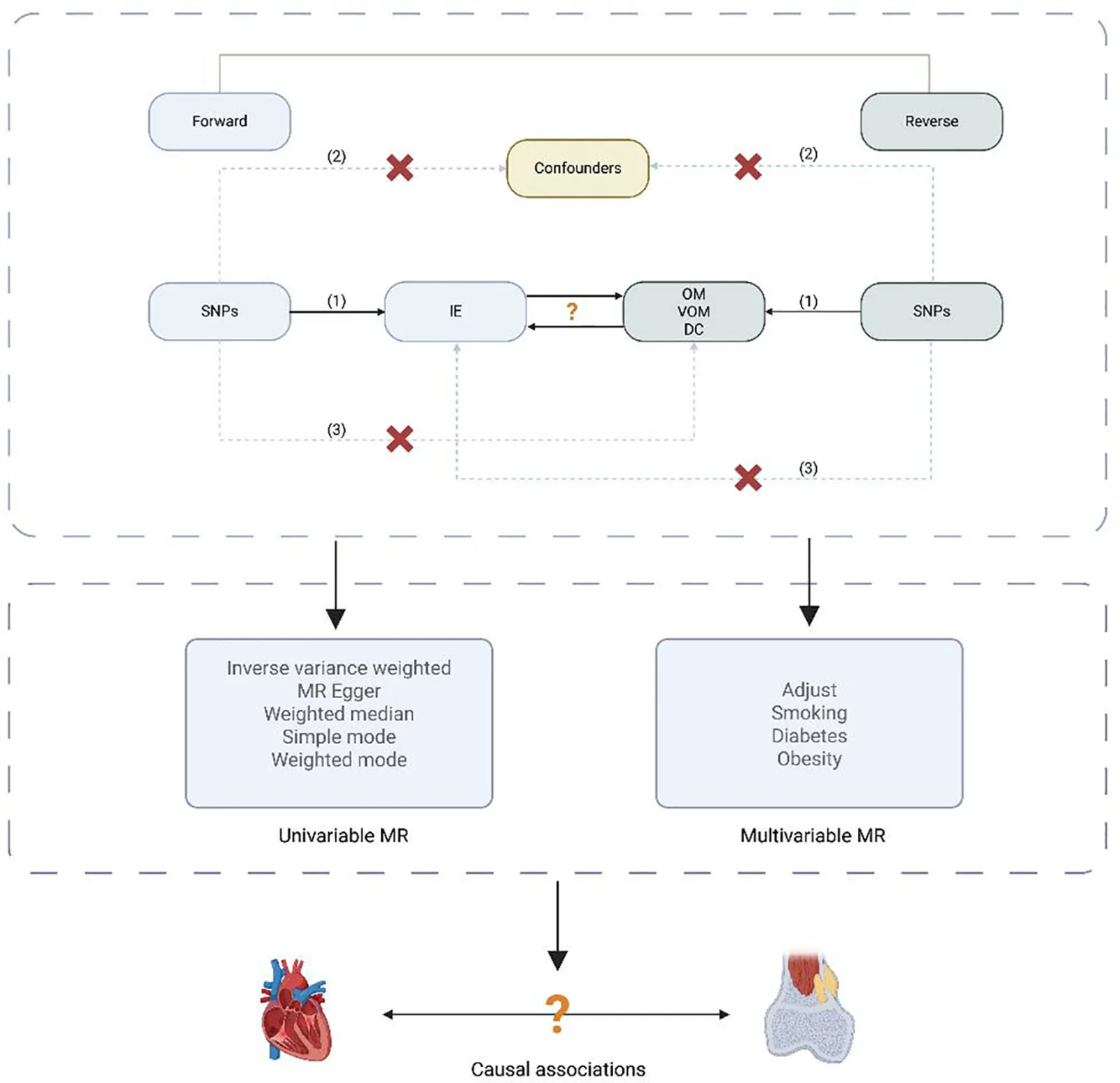
An overview of the design of this Mendelian randomization study: (1) relevance assumption, (2) independence assumption, and (3) exclusion restriction. Created in BioRender (2025, https://BioRender.com/udu6cp3).

This investigation implemented bidirectional MR analysis to examine the putative causal interplay between OM and IE, as illustrated in Figure 1. Our analytical framework incorporated univariable and multivariable MR configurations, leveraging consolidated GWAS datasets to quantify exposure–outcome effects (IE→OM). Complementary reverse MR modeling was subsequently executed to delineate potential inverse causal pathways (OM→IE).

To ensure consistency in the ancestral origin of genetic variants and minimize confounding bias, we focused on the largest available dataset of individuals of European ancestry, ensuring that allele frequencies matched between exposure and outcome data. MR was reported with reference to the STROBE-MR guidelines^[19]^.

The IV must satisfy 3 key assumptions: association: the IV should be strongly linked to the risk factor of interest; independence: the IV should not be related to any confounders in the relationship between the risk factor and the outcome; and exclusion restriction: the IV should influence the outcome solely through its effect on the risk factor. Since alleles follow the principle of random allocation, MR studies avoid confounding and reverse causation (common issues in traditional epidemiological research) thus providing a more reliable evaluation of causality.

#### 2.2.2. Data sources

Since discitis (DC) is closely related to VOM, we included both conditions together in our analysis. For IE, we used data published by the UK Biobank through the IEU Open GWAS platform (Project ID: ieu-b-49720, https://gwas.mrcieu.ac.uk), which included a total of 486,484 participants (1080 cases and 485,404 controls). The diagnosis of cases was based on IE ICD codes (ICD-9: 421, 4249, 0932A; ICD-10: I33, I38, I39). GWAS summary data for OM, VOM, and DC in a European population were obtained from the FinnGen project (https://www.finngen.fi/fi). The data for OM included 2336 cases and 473,264 controls, with OM ICD codes (ICD-10 M86, ICD-9 730 [0–9], and ICD-8 720). Data for VOM included 111 cases and 353,224 controls, with the VOM ICD code (ICD-10 M46.2). The data for DC included 557 cases and 353,224 controls, with the ICD code for DC (ICD-10 M46.4).

To enhance the robustness of our results, we selected different data sources for secondary analyses to further validate the reliability of the findings. For the secondary analysis of IE, we used the GWAS summary data from the FinnGen project, which included 1276 cases and 375,343 controls. For OM, we used data from the UK Biobank through the IEU Open GWAS platform (Project ID: ieu-b-4975), with a total of 486,484 participants (4836 cases and 481,648 controls).

Additionally, we included smoking, obesity, and diabetes in the multivariable analyses due to their potential immunosuppressive effects.^[20–22]^ By adjusting for these 3 confounders, we aimed to further clarify the causal relationship between IE and OM. The data sources for these confounders were all obtained from the UK Biobank via the IEU Open GWAS platform: smoking (item ID: ukb-b-223, total sample size 462,434 participants), obesity (item ID: ukb-b-15541, total sample size 463,010 participants), and diabetes mellitus (item ID: ukb-b-10753, total sample size 463,010 participants). For further details on the data sources, please refer to Supplementary Material S3, Supplemental Digital Content, https://links.lww.com/MD/Q27.

#### 2.2.3. Instrumental variables selection and data harmonization

To ensure IV complied with MR assumptions (association, independence, and exclusivity), we conducted quality control steps: selected exposure-associated single nucleotide polymorphisms (SNPs) using adjusted genome-wide thresholds (*P* < 5 × 10⁻⁶ after initial 5 × 10⁻⁸ screening yielded no variants). Performed LD clustering (*R*² < 0.01, 10,000 kb window) to eliminate redundant variants. Applied LDlink analysis to identify and remove SNPs associated with key confounders including smoking, diabetes, and autoimmune disorders. Harmonized exposure–outcome datasets by excluding palindromic SNPs and alleles with mismatched effects, followed by validation through *R*² calculations and *F*-statistic filtering (*F* ≥ 10 threshold for IV strength).

#### 2.2.4. Statistical analysis

The primary causal effects were analyzed using IVW estimation, which provides optimal precision when valid instruments are available.^[23]^ Sensitivity analyses included MR-Egger regression (assessing directional pleiotropy), MR-PRESSO outlier detection, and Cochran *Q* test for heterogeneity. Leave-one-out analysis verified result stability.

Multivariable MR (MVMR) adjusted for diabetes, obesity, and smoking by analyzing overlapping SNPs across 4 traits, reducing confounding bias. Following quality control, MVMR analysis generated robust estimates under varying pleiotropy conditions.

All analyses were implemented in R v4.4.2 using Two-Sample MR (univariable MR) and MVMR packages, with statistical significance defined as 2-sided *P* < .05.

## 3. Results

### 3.1. Meta-analysis of prevalence

#### 3.1.1. Literature search process

A total of 3890 documents were retrieved, and 2473 documents remained after removing 1417 duplicates. Based on the exclusion criteria, the initial screening, which involved reviewing the title and abstract, resulted in 51 potential relevant documents. After full-text evaluation, 28 papers were excluded for various reasons: 2 studies were case reports^[24,25]^; 13 studies lacked relevant data^[26–38]^; and 13 studies had a sample size of fewer than 100 participants.^[39–51]^ To address potential publication bias, we implemented a dual-strategy approach: systematic backward citation tracking of all included studies’ reference lists, and forward literature surveillance through PubMed alerts and preprint monitoring. This comprehensive retrieval strategy identified 7 additional qualifying studies (4 from citation mining and 3 from emerging evidence sources) that satisfied our predefined inclusion parameters.^[52–57]^ Ultimately, 30 relevant papers were included for analysis.^[16,34,42,52–78]^ The complete literature screening process is outlined in Supplementary Material S4, Supplemental Digital Content, https://links.lww.com/MD/Q27.

#### 3.1.2. Basic characteristics of the included literature

A total of 30 studies were included in the analysis^[16,34,42,52–70,72–78]^, comprising 5 prospective studies,^[34,57,69,73,78]^ and 25 retrospective studies.^[16,42,52–56,58–68,70,72,74–77]^ The retrospective studies included retrospective cohort studies, case–control studies, and case series. The combined sample size for analysis was 32,083. The specific quality assessment methods for the included studies are detailed in Supplementary Material S5, Supplemental Digital Content, https://links.lww.com/MD/Q27.

#### 3.1.3. Concomitant conditions of both IE and OM

**Figure 2.**
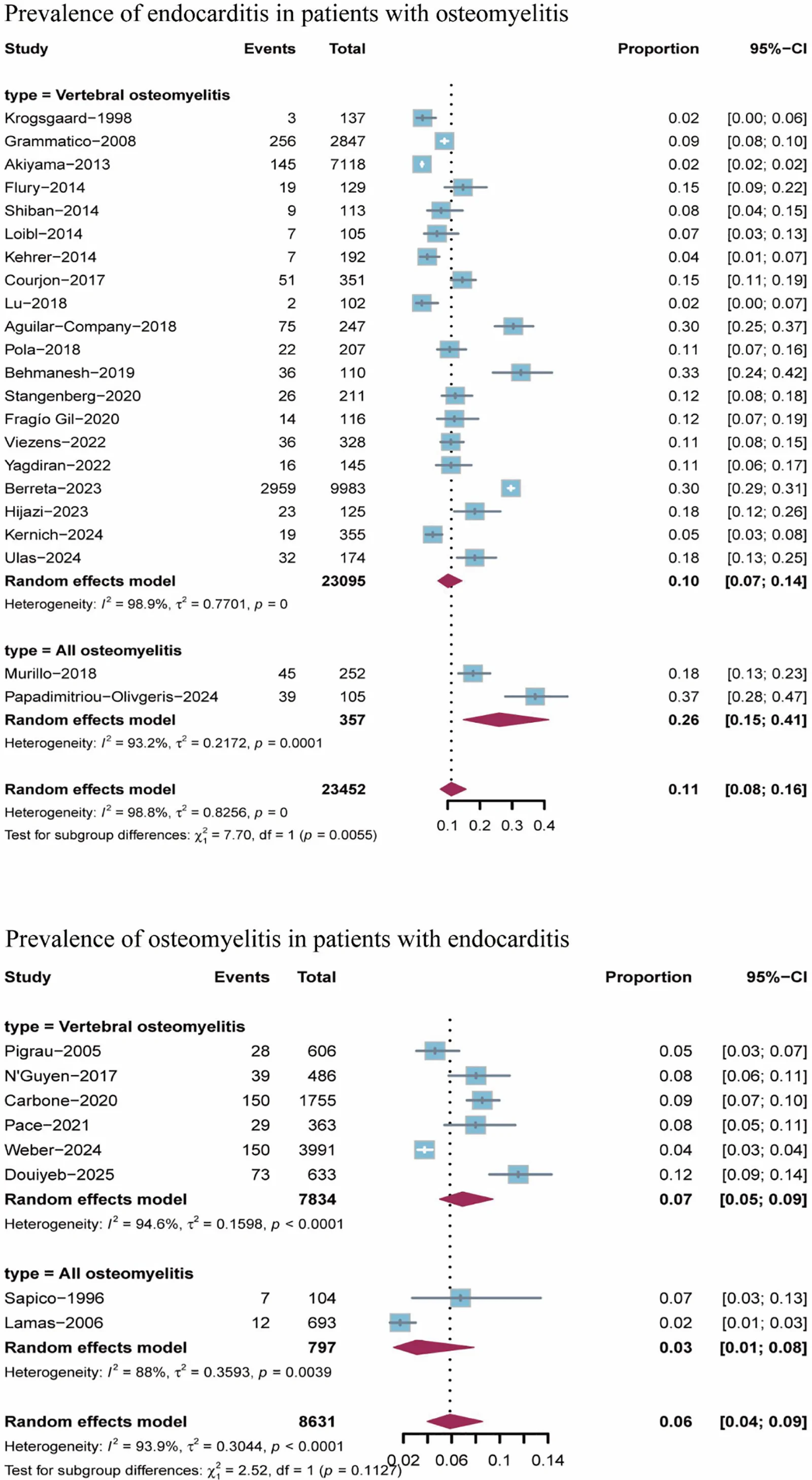
Results of meta-analysis.

A total of 22 studies examined the co-occurrence of IE in patients with OM. The analysis revealed that the prevalence of IE among patients with OM was 11% (95% CI = 8–16%). Additionally, 8 studies assessed the prevalence of OM in patients with IE, with the analysis showing that the prevalence of OM in patients with IE was 6% (95% CI = 4–9%) (see Fig. 2).

#### 3.1.4. Sensitivity analysis

We conducted a sensitivity analysis using the metainf() function, systematically excluding 1 study at a time to assess its impact on the pooled results. The findings showed that excluding any individual study did not lead to a significant change in the rate of IE complications in patients with OM, nor did it affect the rate of OM complications in patients with IE. Further details are provided in Supplementary Material S6, Supplemental Digital Content, https://links.lww.com/MD/Q27.

#### 3.1.5. Publication bias

The Egger test was applied using the metabias() function to assess publication bias for each indicator. The results indicated no significant publication bias for the prevalence of IE in patients with OM (*P* = .8919). However, a potential publication bias was detected in the prevalence of OM in patients with IE (*P* = .0213).

### 3.2. MR

#### 3.2.1. IV obtained according to the quality control process

We selected IV with *P*-values < 5 × 10⁻⁶ and LD *R*² > 0.001. Additionally, confounder SNPs were identified using LDlink (https://ldlink.nih.gov/?tab=home) and manually excluded. The selected SNPs had *F*-statistics >10, and the effect estimates for the association between each SNP and IE, OM, VOM, and DC are reported in Supplementary Material S7, Supplemental Digital Content, https://links.lww.com/MD/Q27.

#### 3.2.2. MR results for IE and OM, VOM, and DC

**Figure 3.**
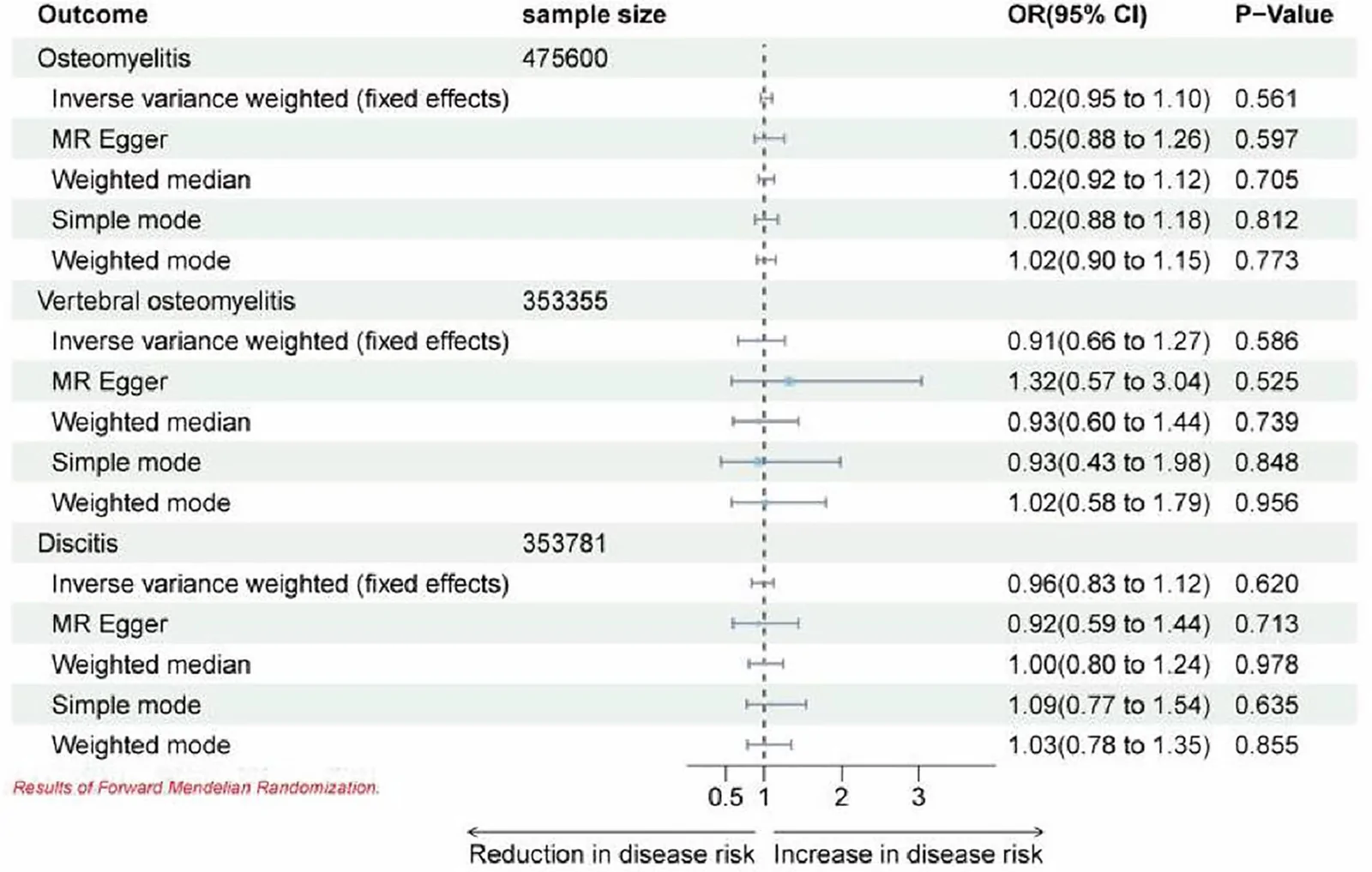
Results of the initial forward Mendelian randomization analysis. DC = discitis; IE = infective endocarditis; OM = osteomyelitis; VOM = vertebral osteomyelitis.

**Figure 4.**
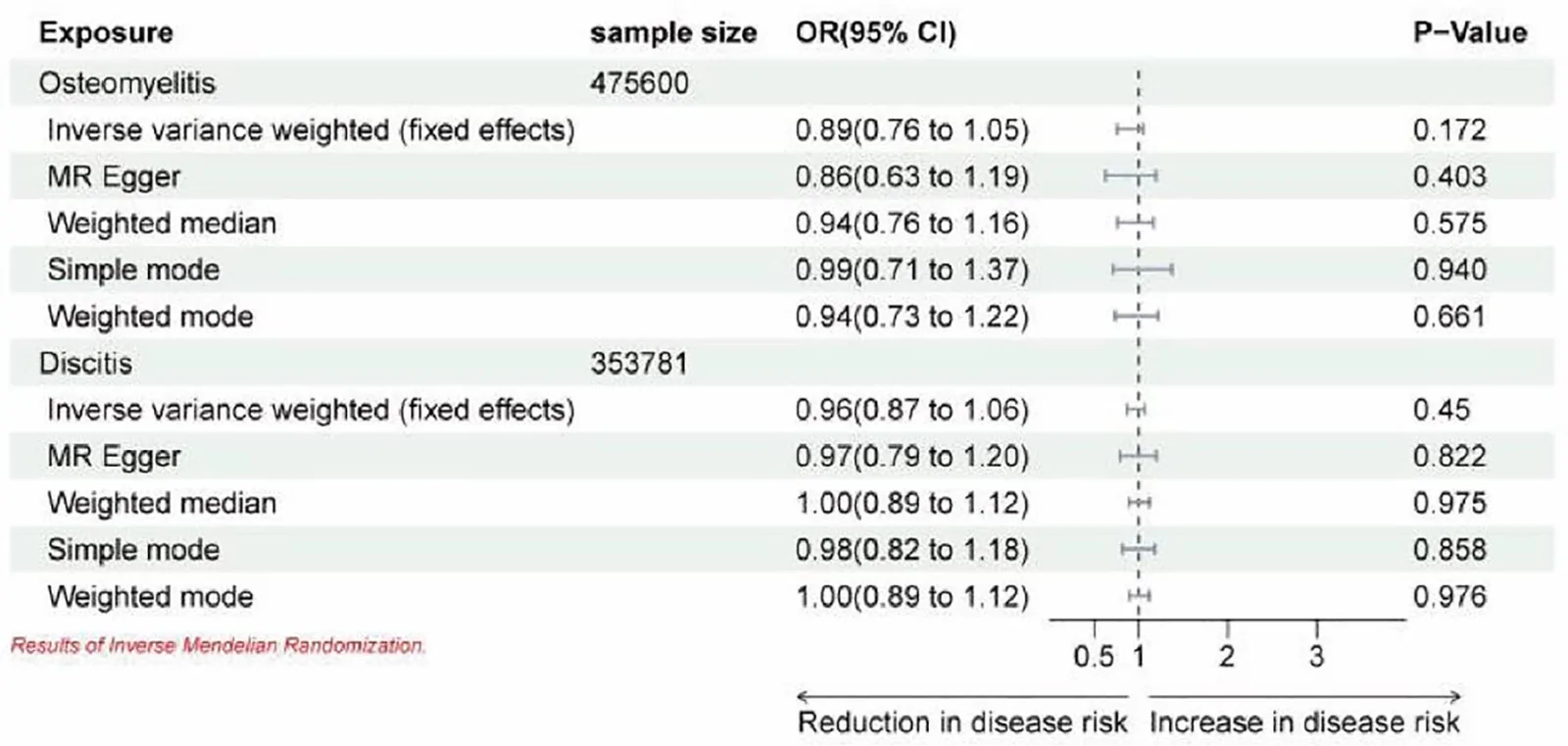
Results of the initial reverse Mendelian randomization analysis. DC = discitis; IE = infective endocarditis; OM = osteomyelitis.

Figure 2 shows the MR estimates for the risk of IE in relation to OM, VOM, and DC. The forward MR analysis yielded the following results: IE → OM (IVW odds ratios [OR]: 1.02; 95% CI = 0.95–1.10, *P* = .561). IE → VOM (IVW OR: 0.91; 95% CI = 0.66–1.27, *P* = .586). IE → DC (IVW OR: 0.96; 95% CI = 0.83–1.12, *P* = .620). The reverse MR analysis showed: OM → IE (IVW OR: 0.89; 95% CI = 0.76–1.05, *P* = .172). DC → IE (IVW OR: 0.96; 95% CI = 0.87–1.06, *P* = .450). When VOM was used as exposure, no SNPs were acquired after the quality control process, so this analysis was not further pursued. Consistent results across various methods, including IVW, weighted median, MR-Egger regression, simple model, and weighted model, showed *P*-values >.05 (see Figs. 3 and 4). Differences in analysis platforms, experiments, and populations may contribute to variability in IVs, which could introduce heterogeneity affecting MR analysis results. Heterogeneity was assessed using the IVW and MR-Egger intercept methods, with all *P*-values >.05, indicating low likelihood of heterogeneity in this study (see Supplementary Material S8, Supplemental Digital Content, https://links.lww.com/MD/Q27).

We further validated the causal relationship between IE and OM using GWAS data from different sources, yielding the following results: forward MR IVW (OR: 0.99; 95% CI = 0.91–1.08, *P* = .853). Reverse MR IVW (OR: 0.79; 95% CI = 0.58–1.08, *P* = .140) (see Supplementary Material S9, Supplemental Digital Content, https://links.lww.com/MD/Q27).

In separate multivariate MR analyses for IE and OM, adjusting for genetically predicted smoking, diabetes, and obesity, the OR for IE→OM in the IVW analysis was 0.95 (95% CI = 0.75–1.20, *P* = .646). Detailed information is provided in Supplementary Material S10, Supplemental Digital Content, https://links.lww.com/MD/Q27. In summary, none of the results in the MR analyses showed a significant causal link.

#### 3.2.3. Pleiotropy and sensitivity analysis

Both horizontal pleiotropy tests (MR-Egger intercept *P* > .05; MR-PRESSO global test *P* > .05) confirmed minimal bias, reinforcing result validity (see Supplementary Material S8, Supplemental Digital Content, https://links.lww.com/MD/Q27). Leave-one-out sensitivity analysis demonstrated stable causal estimates across all SNP exclusions (see Supplementary Material S11, Supplemental Digital Content, https://links.lww.com/MD/Q27).

#### 3.2.4. Meta-analytic merging of 2-sample MR IVW results

The OR and 95% CI for the 2-sample MR IVW method, combining IE and OM, were analyzed using meta-analysis. The results were as follows: forward MR IVW (OR: 1.01; 95% CI = 0.96–1.07). Reverse MR IVW (OR: 0.87; 95% CI = 0.75–1.00) (see Supplementary Material S12, Supplemental Digital Content, https://links.lww.com/MD/Q27).

## 4. Discussion

This study investigated the causal relationship between IE and OM. Meta-analysis revealed that the prevalence of IE among patients with OM was 11% (95% CI = 8–16%), while the prevalence of OM among patients with IE was 6% (95% CI = 4–9%). However, MR analysis indicated that the causal association between IE and OM was not strong. Nonetheless, the findings of this study require further interpretation.

The potential link between OM and IE has been recognized for some time. Many researchers have highlighted the importance of considering the coexistence of these 2 conditions during diagnosis and treatment, particularly in patients with VOM.^[51,79]^ Existing research indicates that hematogenous dissemination of microorganisms from cardiac lesions might serve as a pathogenetic pathway for spinal infection development, particularly VOM.^[68]^ Given that the vertebral body is supplied by a dense network of arteries, bloodstream transmission is thought to be a key mode of infection.^[80]^ Relevant guidelines also emphasize that clinicians should remain vigilant for VOM in patients with IE.^[7]^ These studies suggest a strong association between OM, particularly VOM, and IE.

However, the MR analysis, which primarily utilized data from the UK Biobank and FinnGen, did not yield the expected results. Specifically, no significant differences were observed across different data sources. This contradictory outcome should be interpreted with caution.

MR, as a novel approach, can mimic randomized controlled trials and effectively control for confounding factors, thereby providing stronger evidence of causal associations.^[81,82]^ However, this method has its limitations, as it primarily reflects lifetime cumulative genetic risk exposures, making it challenging to capture short-term or dynamic pathological processes.^[83]^ Specifically, the transient and rapid onset of OM due to bloodstream transmission of pathogenic bacteria that cause IE may elevate the short-term risk of OM in patients with IE. However, MR is less effective in capturing such acute events. This limitation may explain why MR failed to demonstrate the expected results, despite the strong clinical association between OM and IE. Moreover, IE and OM are primarily infectious diseases triggered by environmental exposures, particularly pathogenic microbial infections. Genetic factors may not play a dominant role in the onset of these diseases but are more likely to influence the host’s susceptibility to infection, the severity of the infection, and the risk of complications such as bacteremia through indirect pathways.

The close association between bacteremia, OM, and IE has been reported in numerous studies.^[84–86]^ In a study by Koslow et al, it was found that gram-positive bacteremia occurred more frequently in patients with co-infected IE than in those with VOM alone.^[47]^ The authors suggested that VOM is likely a complication of infected IE. This indicates that bacteremia may play a mediating role in the relationship between IE and OM, potentially mediating the causal link between the 2 conditions. Confounding factors may also partially explain the observed association, but they do not establish a causal relationship between IE and OM. Both IE and OM are multifactorial, complex diseases that involve prolonged exposure to various risk factors. As a result, their association may be influenced by shared confounders. Key risk factors such as smoking, obesity, and diabetes play a significant role in the development of both conditions.^[87–89]^

Therefore, our negative MR results lead to the following insights: exclusion of a strong genetic causal pathway: there may not be a direct causal chain driven by common, strong lifelong genetic risk factors between IE and OM. Support for a non-genetic dominant mechanism: the strong clinical association observed between IE and OM (as demonstrated by the significant co-occurrence rates in the meta-analysis) is more likely to be primarily or entirely driven by non-genetic factors, with bacteremia playing a central mediating role in the pathophysiology. IE is commonly accompanied by persistent bacteremia, which is the primary pathogenesis of hematogenous disseminated OM, such as VOM. *S aureus*, a major common pathogen of both, has virulence characteristics (such as adhesion molecules and biofilm-forming ability) that allow it to colonize damaged endocardium and form vegetations, while also spreading to the bones (particularly the richly vascularized vertebral bodies) to induce OM.^[90,91]^ The clinical co-occurrence typically reflects simultaneous infection of different target organs caused by the same severe infectious event (such as bacteremia). For example, IE and OM may exist as concurrent complications, or in the same patient, sustained bacteremia may cause IE to lead to OM, or OM to trigger IE. The co-occurrence of these complications is primarily related to the spread of infection rather than shared genetic backgrounds. Clinical guidelines recommend screening for VOM in IE patients precisely based on the profound understanding of this bacteremia-mediated pathological link.

This study has some limitations. First, the results of the meta-analysis exhibited significant heterogeneity, which may be attributed to 3 key factors: variations in the inclusion criteria for OM across studies, differences in the types of OM considered, and the varying ability of different causative organisms to induce OM. Second, to obtain appropriate IV, we selected a relatively low *P*-value threshold of 5 × 10⁻⁶. Additionally, we did not adjust for covariates such as age, gender, and others in the 2-sample MR analysis. Finally, the current whole-genome sequencing data primarily focus on European populations, while gene frequencies may vary across different populations. As a result, these findings should be interpreted with caution when applied to populations from other regions.

However, there are notable strengths to this study. First, the meta-analysis process adhered to the Preferred Reporting Items for Systematic Reviews and Meta-Analyses guidelines, and we manually screened references for relevant studies to minimize publication bias. Second, the MR analysis was conducted following the STROBE-MR guidelines, and the association between IE and OM was further validated through multivariate MR and data from multiple sources. Lastly, although the 2-sample MR did not adjust for covariates, the heterogeneity, sensitivity, and multiplicity analyses throughout the study produced satisfactory results, indicating the robustness of our findings. These strengths contribute to the reliability and transparency of our study.

This study provides a deeper understanding of the association between IE and OM. Although the MR results did not show a significant causal association, this does not diminish the strong clinical relationship observed between the 2 conditions. The role of bacteremia in the association between IE and OM warrants further investigation to better clarify this link.

## 5. Conclusion

Despite the statistically negative results from MR, the close association between IE and OM remains evident. Future research should explore the mediating role of bacteremia in this association and incorporate genetic data from diverse ethnic cohorts and dynamic time dimensions to more fully elucidate the complex relationship between IE and OM. In clinical practice, based on the available evidence, vigilance is essential for patients with comorbid OM and IE, particularly in the screening and treatment of VOM and IE, where the pathogenic mechanism of blood-borne transmission continues to serve as a crucial guide, as genetic factors may not play a dominant role in the development of these diseases.

## Author contributions

**Conceptualization:** Huancai Liu, Zhengqi Chang.

**Data curation:** Xue Yu, Yanan Wang.

**Investigation:** Huan Liu, Hao Xing.

**Writing – original draft:** Huan Liu.

**Writing – review & editing:** Huancai Liu, Zhengqi Chang.

## Supplementary Material
