# Supplementary material for: Two-sample Mendelian randomization investigating the causal association between endocarditis and osteomyelitis: A validation analysis using UK Biobank and FinnGen databases
Source: Medicine (Baltimore). 2025 Sep 26;104(39):e44651. doi: 10.1097/MD.0000000000044651 (PMC13593316; doi:10.1097/MD.0000000000044651)
Supplement: Supplementary file 1 [file medi-104-e44651-s001.pdf]

Supplementary Material 1: Search History

1.PubMed

Search number	Query	Results
1	"Endocarditis"[Mesh]	31,642
2	(((((Endocarditis[Title/Abstract]) OR (Endocarditides[Title/Abstract])) OR (Infective Endocarditis[Title/Abstract])) OR (Endocarditides, Infective[Title/Abstract])) OR (Endocarditis, Infective[Title/Abstract])) OR (Infective Endocarditides[Title/Abstract])	42,452
3	("Endocarditis"[Mesh]) OR ((((((Endocarditis[Title/Abstract]) OR (Endocarditides[Title/Abstract])) OR (Infective Endocarditis[Title/Abstract])) OR (Endocarditides, Infective[Title/Abstract])) OR (Endocarditis, Infective[Title/Abstract])) OR (Infective Endocarditides[Title/Abstract]))	47,604
4	"Osteomyelitis"[Mesh]	25,255
5	(Osteomyelitis[Title/Abstract]) OR (Osteomyelitides[Title/Abstract])	28,520
6	("Osteomyelitis"[Mesh]) OR ((Osteomyelitis[Title/Abstract]) OR (Osteomyelitides[Title/Abstract]))	36,629
7	(("Osteomyelitis"[Mesh]) OR ((Osteomyelitis[Title/Abstract]) OR (Osteomyelitides[Title/Abstract]))) AND (("Endocarditis"[Mesh]) OR ((((((Endocarditis[Title/Abstract]) OR (Endocarditides[Title/Abstract])) OR (Infective Endocarditis[Title/Abstract])) OR (Endocarditides, Infective[Title/Abstract])) OR (Endocarditis, Infective[Title/Abstract])) OR (Infective Endocarditides[Title/Abstract])))	1,121

2.Embase

Search number	Query	Results
1	'endocarditis'/exp	65,637
2	'endocarditis':ab,ti	56,084

3	'endocarditides':ab,ti	11
4	'infective endocarditis':ab,ti	22,162
5	'endocarditides, infective':ab,ti	0
6	'endocarditis, infective':ab,ti	49
7	'infective endocarditides':ab,ti	0
8	#1 OR #2 OR #3 OR #4 OR #5 OR #6 OR #7	74,271
9	'osteomyelitis'/exp	55,293
10	'osteomyelitis':ab,ti	34,199
14	14	14
12	#10 OR #11	34,206
13	#8 AND #12	1,611

3.Cochrane library

Search number	Query	Results
1	MeSH descriptor: [Osteomyelitis] explode all trees	210
2	(Osteomyelitis):ti,ab,kw OR (Osteomyelitides):ti,ab,kw	743
3	#1 or #2	750
4	MeSH descriptor: [Endocarditis] explode all trees	243
5	(Endocarditis):ti,ab,kw OR (Endocarditides):ti,ab,kw OR (Infective Endocarditis):ti,ab,kw OR (Endocarditides, Infective):ti,ab,kw OR (Endocarditis, Infective):ti,ab,kw	853
6	#4 or #5	853
7	#3 and #6	32

4.Web of science

Search number	Query	Results
1	(TS=(Osteomyelitides)) OR TS=(Osteomyelitis)	28988

2	(((((TS=(Endocarditis)) OR TS=(Endocarditides)) OR TS=(Infective Endocarditis)) OR TS=(Endocarditides, Infective)) OR TS=(Endocarditis, Infective)) OR TS=(Infective Endocarditides)	44994
3	#1 AND #2	1126

Supplementary Material 2: Characteristics of the included studies

Studies examining the incidence of infective endocarditis in patients with osteomyelitis.

Study	Country	Design	No. of patients with IE	Sample size	Types of osteomy -elitis
Krogsgaard1998	Denmark	Retrospective	3	137	Acute vertebral osteomyelitis
Grammatico2008	France	Retrospective	256	2847	Vertebral osteomyelitis
Akiyama2013	Japan	Retrospective	145	7118	Vertebral osteomyelitis
Flury2014	Switzerland	Retrospective	19	129	Primary bacterial vertebral osteomyelitis
Shiban2014	Germany	Retrospective	9	113	Spondylodiscitis
Loibl2014	Germany	Retrospective	7	105	Vertebral osteomyelitis
Kehrer2014	Denmark	Retrospective	7	192	Pyogenic spondylodiscitis
Courjon2017	France	Retrospective	51	351	Pyogenic vertebral osteomyelitis
Lu2018	China	Retrospective	2	102	Infectious spondylodiscitis in hemodialysis patients
Aguilar-Company2018	Spain	Retrospective	75	247	Native vertebral osteomyelitis
Murillo2018	Spain	Retrospective	45	252	Vertebral osteomyelitis and peripheral osteomyelitis
Pola2018	Italy	Retrospective	22	207	Pyogenic spondylodiscitis

Behmanesh2019	Germany	Prospective	36	110	Pyogenic spondylodiscitis
Stangenberg2020	Germany	Retrospective	26	211	Spondylodiscitis
Fragío Gil2020	Spain	Retrospective	14	116	Vertebral osteomyelitis
Viezens2022	Germany	Prospective	36	328	Spontaneous spondylodiscitis
Yagdiran2022	Germany	Retrospective	16	145	Vertebral osteomyelitis
Berreta2023	USA	Retrospective	2959	9983	Spondylodiscitis
Hijazi2023	Germany	Retrospective	23	125	Spondylodiscitis
Kernich2024	Germany	Retrospective	19	355	Vertebral osteomyelitis
Papadimitriou-Olivgeris2024	Switzerland	Retrospective	39	105	Acute osteomyelitis and chronic osteitis
Ulas2024	Netherlands	Prospective	32	174	Spondylodiscitis

Studies related to the incidence of osteomyelitis among patients with infective endocarditis.

Study	Country	Design	No. of patients with OM	Sample size	Types of osteomy -elitis
Sapico1996	USA	Retrospective	7	104	Osteomyelitis
Pigrau2005	Spain	Retrospective	28	606	Spontaneous pyogenic vertebral osteomyelitis
Lamas2006	UK	Retrospective	12	693	Bone infections
N'Guyen2017	France	Retrospective	39	486	Vertebral osteomyelitis
Carbone2020	France	Prospective	150	1755	Spondylodiscitis

Pace2021	Italy	Retrospective	29	363	Spondylodiscitis
Weber2024	Netherlands	Retrospective	150	3991	Vertebral osteomyelitis
Douiyeb2025	Netherlands	Prospective	73	633	Vertebral osteomyelitis

Supplementary Material 3: Summary of the datasets used in the study

Variable	N of cases/ control	Ethnic Ancestry	Link	ICD-code
The initial two-sample Mendelian randomization analysis				
IE	1080/485404	European	ieu-b-49720	ICD-9：421、4249、0932A；ICD10：I33、I38、I39
OM	2336/473264	European	https://storage.googleapis.com/finngen-public-data-r12/summary_stats/release/finngen_R12_M13_OSTEOMYELITIS.gz	ICD-10 M86、ICD-9 730[0-9] and ICD-8 720
VOM	111/353224	European	https://storage.googleapis.com/finngen-public-data-r12/summary_stats/release/finngen_R12_M13_OSTEOMYELVERTEB.gz	ICD-10 M46.2
DC	557/353224	European	https://storage.googleapis.com/finngen-public-data-r12/summary_stats/release/finngen_R12_M13_DISCITIS.gz	ICD-10 M46.4
The secondary two-sample Mendelian randomization analysis				
IE	1276/375343	European	https://storage.googleapis.com/finngen-public-data-r12/summary_stats/release/finngen_R12_I9_ENDOCARD.gz	ICD-9：421、4249、0932A；ICD10：I33、I38、I39
OM	4,836/481648	European	ieu-b-4975	-
Multivariate Mendelian randomization				
IE	1080/485404	European	ieu-b-49720	ICD-9：421、4249、0932A；ICD10：I33、I38、I39
OM	2336/473264	European	https://storage.googleapis.com/finngen-public-data-r12/summary_stats/release/finngen_R12_M13_OSTEOMYELITIS.gz	ICD-10 M86、ICD-9 730[0-9] and ICD-8 720
Smoking	462434	European	ukb-b-223	-
Diabetes	22340/439238	European	ukb-b-10753	-
Obesity	4,688/458,322	European	ukb-b-15541	-

IE:infective endocarditis;OM:osteomyelitis;VOM:vertebral osteomyelitis;DC:discitis.

Supplementary Material 4: Flow of articles through the systematic review

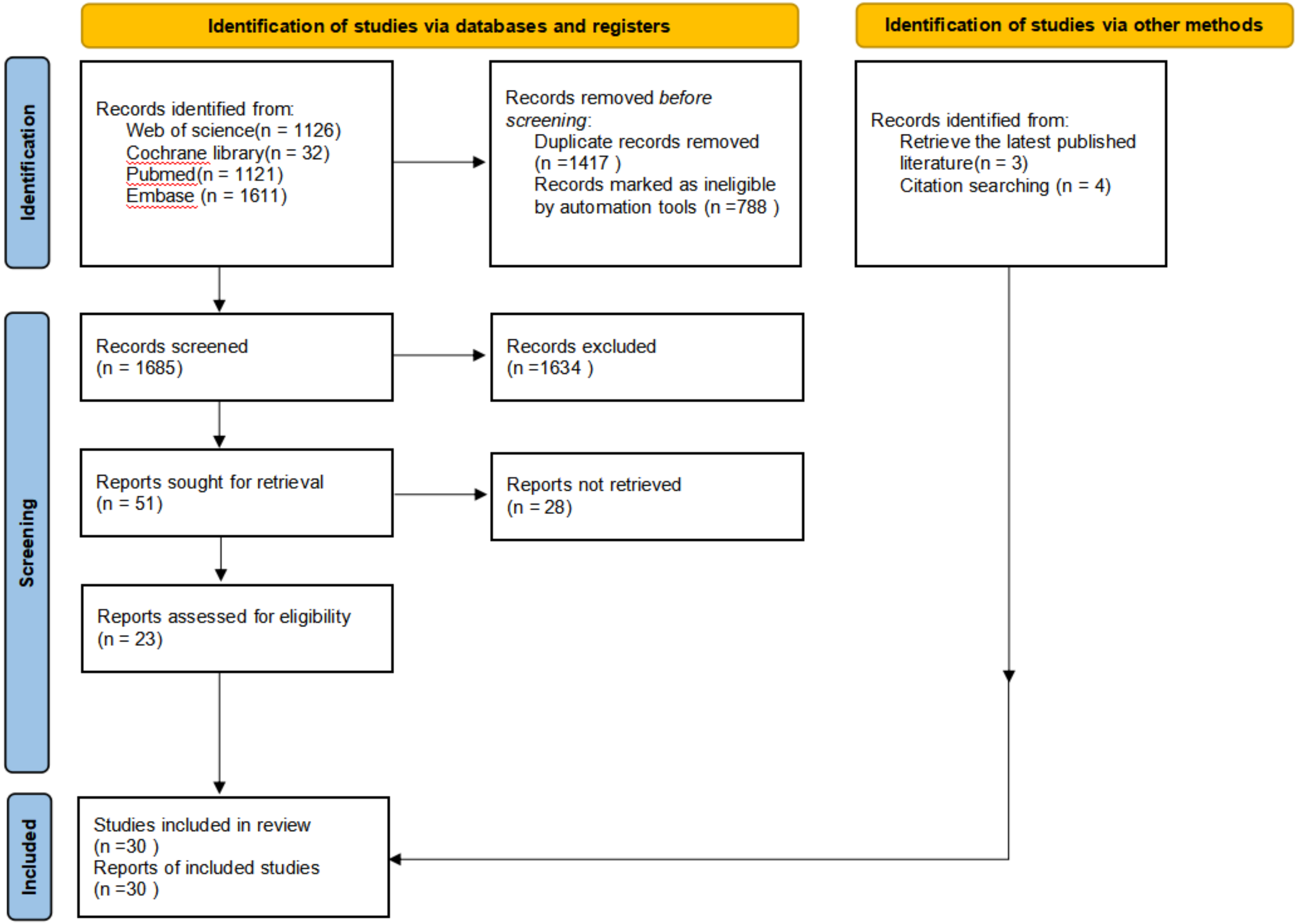

Supplementary Material 5: JBI critical Appraisal Checklist for Study

This meta-analysis did not address disease prognosis, so we did not evaluate the 8th indicator.

Study	(1)	(2)	(3)	(4)	(5)	(6)	(7)	(8)	(9)	(10)
Sapico1996	Y	Y	Y	Y	Y	Y	Y	-	Y	Y
Krogsgaard1998	Y	N	N	Y	N	Y	Y	-	N	Y
Pigrau2005	Y	Y	Y	Y	Y	Y	Y	-	Y	Y
Lamas2006	Y	Y	Y	Y	N	Y	Y	-	Y	Y
Grammatico2008	Y	N	N	Y	Y	Y	Y	-	N	Y
Akiyama2013	Y	N	N	Y	Y	Y	Y	-	Y	Y
Flury2014	Y	N	N	Y	N	Y	Y	-	Y	Y
Shiban014	Y	N	N	Y	Y	Y	Y	-	N	Y
Loibl2014	Y	N	N	Y	Y	Y	Y	-	Y	Y
Kehrer2014	Y	N	N	Y	N	Y	Y	-	Y	Y
Courjon2017	Y	N	N	Y	N	Y	Y	-	N	Y
N'Guyen2017	Y	N	N	Y	Y	Y	Y	-	Y	Y
Lu2018	Y	N	N	Y	Y	Y	Y	-	N	Y
Aguilar- Company2018	Y	Y	Y	Y	Y	Y	Y	-	Y	Y
Murillo2018	Y	Y	Y	Y	U	Y	Y	-	N	Y
Pola2018	Y	N	N	Y	U	Y	Y	-	N	Y
Behmanesh2019	Y	Y	Y	Y	Y	Y	Y	-	N	Y
Carbone2020	Y	Y	Y	Y	Y	Y	Y	-	N	Y
Stangenberg2020	Y	N	N	Y	U	Y	Y	-	N	Y
Fragío Gil2020	Y	N	N	Y	U	Y	Y	-	N	Y
Pace2021	Y	Y	Y	Y	U	Y	Y	-	N	Y
Viezens2022	Y	Y	Y	Y	U	Y	Y	-	N	Y
Yagdiran2022	Y	N	N	Y	N	Y	Y	-	N	Y
Berreta2023	Y	N	N	Y	Y	Y	Y	-	N	Y
Hijazi2023	Y	Y	Y	Y	Y	Y	Y	-	N	Y
Kernich2024	Y	N	N	Y	U	Y	Y	-	N	Y
Papadimitriou-Olivgeris2024	Y	Y	Y	Y	Y	Y	Y	-	N	Y
Ulas2024	Y	Y	Y	Y	Y	Y	Y	-	Y	Y
Weber2024	Y	Y	Y	Y	U	Y	Y	-	Y	Y
Douiyeb2025	Y	Y	Y	Y	Y	Y	Y	-	N	Y

Note: (1) Were there clear criteria for inclusion in the case series?: (2) Was the condition measured in a standard, reliable way for all participants included in the case series?; (3) Were valid methods used for identification of the condition for all participants included in the case series?: (4) Did the case series have consecutive inclusion of participants?: (5) Did the case series have complete inclusion of participants?: (6) Was there clear reporting of the demographics of the participants in the study?: (7) Was there clear reporting of clinical information of the participants?: (8) Were the outcomes or follow up results of cases clearly reported?: (9) Was there clear reporting of the presenting site(s)/clinic(s) demographic information?; (10) Was statistical analysis appropriate? Y: Yes; N: No;U:Unknown

Supplementary Material 6: Sensitivity analysis regarding the incidence rate

Sensitivity analysis of the incidence of endocarditis.

Study	Proportion	95%-CI	P-value	Tau2	Tau	I2
Omitting Krogsgaard-1998	0.12	[0.08; 0.16]	.	0.7494	0.8657	98.9%
Omitting Grammatico-2008	0.11	[0.08; 0.16]	.	0.8693	0.9324	98.7%
Omitting Akiyama-2013	0.12	[0.09; 0.17]	.	0.6597	0.8122	97.4%
Omitting Flury-2014	0.11	[0.08; 0.16]	.	0.8670	0.9311	98.9%
Omitting Shiban-2014	0.11	[0.08; 0.16]	.	0.8583	0.9265	98.9%
Omitting Loibl-2014	0.11	[0.08; 0.16]	.	0.8478	0.9207	98.9%
Omitting Kehrer-2014	0.12	[0.08; 0.16]	.	0.7928	0.8904	98.9%
Omitting Courjon-2017	0.11	[0.08; 0.16]	.	0.8688	0.9321	98.9%
Omitting Lu-2018	0.12	[0.08; 0.16]	.	0.7531	0.8678	98.9%
Omitting Aguilar-Company-2018	0.11	[0.07; 0.15]	.	0.7873	0.8873	98.9%
Omitting Murillo-2018	0.11	[0.07; 0.16]	.	0.8578	0.9262	98.9%
Omitting Pola-2018	0.11	[0.08; 0.16]	.	0.8706	0.9331	98.9%
Omitting Behmanesh-2019	0.10	[0.07; 0.15]	.	0.7763	0.8811	98.9%
Omitting Stangenberg-2020	0.11	[0.08; 0.16]	.	0.8714	0.9335	98.9%
Omitting Fragío Gil-2020	0.11	[0.08; 0.16]	.	0.8696	0.9325	98.9%
Omitting Viezens-2022	0.11	[0.08; 0.16]	.	0.8721	0.9339	98.9%
Omitting Yagdiran-2022	0.11	[0.08; 0.16]	.	0.8699	0.9327	98.9%
Omitting Berreta-2023	0.11	[0.07; 0.15]	.	0.7885	0.8880	97.1%
Omitting Hijazi-2023	0.11	[0.07; 0.15]	.	0.8556	0.9250	98.9%
Omitting Kernich-2024	0.12	[0.08; 0.16]	.	0.8314	0.9118	98.9%
Omitting Papadimitriou-Olivgeris-2024	0.10	[0.07; 0.15]	.	0.7454	0.8634	98.9%
Omitting Ulas-2024	0.11	[0.07; 0.15]	.	0.8557	0.9250	98.9%
**Random effects model**	**0.11**	**[0.08; 0.16]**	**.**	**0.8256**	**0.9086**	**98.8%**

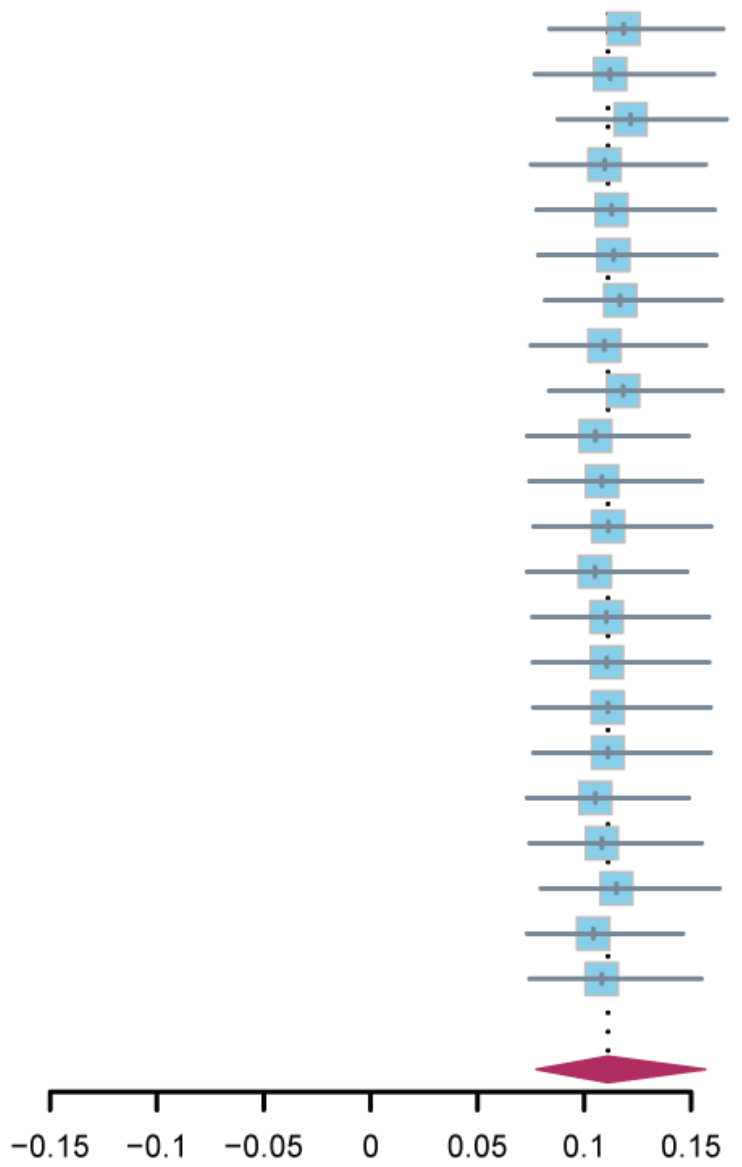

Sensitivity analysis of the incidence of osteomyelitis.

Study	Proportion	95%-CI	P-value	Tau2	Tau	I2
Omitting Sapico-1996	0.06	[0.04; 0.09]	.	0.3409	0.5839	94.7%
Omitting Pigrau-2005	0.06	[0.04; 0.09]	.	0.3419	0.5847	94.6%
Omitting Lamas-2006	0.07	[0.05; 0.09]	.	0.1456	0.3815	93.5%
Omitting N'Guyen-2017	0.06	[0.04; 0.09]	.	0.3396	0.5827	94.6%
Omitting Carbone-2020	0.06	[0.04; 0.08]	.	0.3293	0.5739	93.6%
Omitting Pace-2021	0.06	[0.04; 0.09]	.	0.3399	0.5830	94.7%
Omitting Weber-2024	0.06	[0.04; 0.09]	.	0.3105	0.5572	88.2%
Omitting Douiyeb-2025	0.05	[0.04; 0.08]	.	0.2565	0.5064	92.6%
**Random effects model**	**0.06**	**[0.04; 0.09]**	**.**	**0.3044**	**0.5518**	**93.9%**

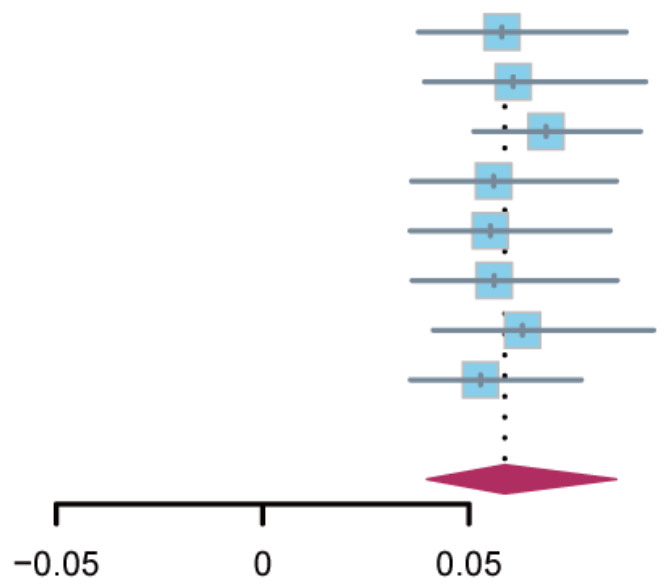

Supplementary Material 7: Detailed information on the instrumental variables used in Mendelian randomization analysis to explore the effects between infective endocarditis and osteomyelitis, vertebral osteomyelitis, and discitis.

Initial forward two-sample Mendelian randomization

SNP	Effect Allele	Other Allele	Association with IE					Associated with OM, VO, and DC			
			EAF	Beta	S.E	P value	F-statistic	EAF	Beta	S.E	P value
IE→OM											
rs147965016	T	C	0.0168784	0.85232	0.179519	2.06E-06	22.54149587	0.0261545	0.00978645	0.0945214	0.917537
rs17127649	T	C	0.0754615	0.420388	0.0849683	7.51E-07	24.47850991	0.0900065	-0.00217866	0.0514907	0.96625
rs1826374	C	G	0.266645	-0.243437	0.0500434	1.15E-06	23.66343418	0.254957	-0.0358621	0.03379	0.288543
rs190727057	A	G	0.0358035	-0.581311	0.125026	3.33E-06	21.61795572	0.113282	-0.0150747	0.0472078	0.749478
rs2825712	A	T	0.111662	-0.333401	0.0708098	2.50E-06	22.16895773	0.0968024	-0.0891094	0.0500314	0.0749014
rs62001061	C	G	0.139517	-0.30739	0.0639778	1.55E-06	23.08442602	0.0919835	0.0510107	0.0509504	0.316738
rs62376596	A	G	0.257496	0.235019	0.051248	4.52E-06	21.03053495	0.252047	0.0170033	0.034007	0.617078
rs6560228	A	C	0.846805	-0.286191	0.0618502	3.71E-06	21.41055584	0.871019	-0.0130179	0.0441277	0.767989
rs73788211	T	G	0.0180376	-0.760628	0.162954	3.05E-06	21.78776993	0.0160715	0.025465	0.115558	0.825587
rs75163228	C	G	0.0354407	0.572723	0.124406	4.15E-06	21.19360375	0.0329434	0.0102754	0.0837564	0.902359
rs78224152	C	T	0.0933156	-0.350008	0.0759136	4.01E-06	21.25763678	0.100185	-0.00510703	0.0490856	0.917135
rs80160614	T	C	0.021741	-0.699277	0.151245	3.77E-06	21.37640407	0.0111467	-0.155547	0.142496	0.275015

rs9519915	C	T	0.382378	-0.236017	0.0455639	2.22E-07	26.83137975	0.323503	0.0549594	0.0315665	0.0816714
IE→VOM											
rs147965016	T	C	0.0168784	0.85232	0.179519	2.06E-06	22.5414958664139	0.0259123	-0.0946531	0.432134	0.826622
rs17127649	T	C	0.0754615	0.420388	0.0849683	7.51E-07	24.4785099088531	0.0903024	0.0394997	0.23404	0.865975
rs1826374	C	G	0.266645	-0.243437	0.0500434	1.15E-06	23.6634341843941	0.255447	-0.0332465	0.153291	0.828298
rs190727057	A	G	0.0358035	-0.581311	0.125026	3.33E-06	21.6179557216713	0.112494	-0.0891418	0.214262	0.677381
rs2825712	A	T	0.111662	-0.333401	0.0708098	2.50E-06	22.1689577297983	0.0978511	0.0785896	0.223152	0.724705
rs62001061	C	G	0.139517	-0.30739	0.0639778	1.55E-06	23.084426018769	0.0922214	0.0515295	0.233778	0.825544
rs62376596	A	G	0.257496	0.235019	0.051248	4.52E-06	21.0305349530262	0.252484	-0.0656366	0.155132	0.672222
rs6560228	A	C	0.846805	-0.286191	0.0618502	3.70706328384e-06	21.4105558430551	0.870448	0.0203273	0.199388	0.918797
rs73788211	T	G	0.0180376	-0.760628	0.162954	3.05E-06	21.7877699259675	0.0159789	0.678557	0.533807	0.20367
rs75163228	C	G	0.0354407	0.572723	0.124406	4.15E-06	21.1936037452564	0.0331023	0.535707	0.375551	0.153737
rs78224152	C	T	0.0933156	-0.350008	0.0759136	4.01E-06	21.2576367784224	0.10034	0.358165	0.221791	0.106338
rs80160614	T	C	0.021741	-0.699277	0.151245	3.77E-06	21.3764040690179	0.0111624	-0.65667	0.663856	0.322578
rs9519915	C	T	0.382378	-0.236017	0.0455639	2.22E-07	26.8313797532236	0.323122	0.162092	0.14322	0.257732
IE→DC											

rs147965016	T	C	0.0168784	0.85232	0.179519	2.06E-06	22.5414958664139	0.0259219	0.233026	0.191903	0.224638
rs17127649	T	C	0.0754615	0.420388	0.0849683	7.51E-07	24.4785099088531	0.0902969	-0.0370597	0.105342	0.724987
rs1826374	C	G	0.266645	-0.243437	0.0500434	1.15E-06	23.6634341843941	0.255429	-0.0647195	0.0689686	0.348044
rs190727057	A	G	0.0358035	-0.581311	0.125026	3.33E-06	21.6179557216713	0.1125	0.0291516	0.0962664	0.762025
rs2825712	A	T	0.111662	-0.333401	0.0708098	2.50E-06	22.1689577297983	0.097834	-0.0764315	0.103319	0.459445
rs62001061	C	G	0.139517	-0.30739	0.0639778	1.55E-06	23.084426018769	0.0922026	-0.123232	0.104823	0.239749
rs62376596	A	G	0.257496	0.235019	0.051248	4.52E-06	21.0305349530262	0.252494	0.0248517	0.0693148	0.719944
rs6560228	A	C	0.846805	-0.286191	0.0618502	3.71E-06	21.4105558430551	0.870447	-0.00692927	0.0901551	0.938735
rs73788211	T	G	0.0180376	-0.760628	0.162954	3.05E-06	21.7877699259675	0.0159827	0.29934	0.241376	0.214924
rs75163228	C	G	0.0354407	0.572723	0.124406	4.15E-06	21.1936037452564	0.0330927	-0.121081	0.166571	0.467284
rs78224152	C	T	0.0933156	-0.350008	0.0759136	4.01E-06	21.2576367784224	0.100326	-0.0124618	0.100512	0.901329
rs80160614	T	C	0.021741	-0.699277	0.151245	3.77E-06	21.3764040690179	0.0111755	0.575845	0.283299	0.0420891
rs9519915	C	T	0.382378	-0.236017	0.0455639	2.22E-07	26.8313797532236	0.323163	0.147239	0.0643048	0.0220389

Initial reverse two-sample Mendelian randomization

**SNP**	**Effect Allele**	**Other Allele**	**Association with OM and DC**					**Associated with IE**			
			**EAF**	**Beta**	**S.E**	**P value**	**F-statistic**	**EAF**	**Beta**	**S.E**	**P value**

OM→IE											
rs115025936	G	A	0.0162011	0.493645	0.0995804	7.15E-07	24.5742304050044	0.0127817	0.0257548	0.202527	0.898808353
rs115197623	G	A	0.0415176	0.308639	0.066913	3.98E-06	21.2754510838473	0.0283792	0.0422407	0.132738	0.750313914
rs116356993	T	C	0.0171527	0.486511	0.099414	9.89E-07	23.9490563166538	0.0159096	0.159405	0.176544	0.366569224
rs146036120	G	A	0.0140536	-0.712893	0.155106	4.30E-06	21.1246575935377	0.00958752	0.27855	0.241299	0.248345903
rs1829559	C	T	0.0192093	0.432303	0.0931235	3.45E-06	21.5504375211953	0.0222086	-0.169141	0.15056	0.261261851
rs204993	G	A	0.180261	0.197776	0.0363951	5.51E-08	29.5297328408937	0.31775	-0.0129266	0.0475732	0.785837953
rs3806155	T	A	0.0542515	0.44425	0.057074	7.04E-15	60.5865781537745	0.0345011	-0.220565	0.120249	0.0666192900223
rs392557	C	A	0.0657312	-0.305983	0.0649242	2.44E-06	22.2115853053524	0.067153	-0.00105611	0.0894537	0.990580216
rs72693421	C	A	0.00535713	0.721587	0.157049	4.33E-06	21.1108258797946	0.0308618	-0.100337	0.131498	0.445441832
rs73052629	G	C	0.00936457	-0.95539	0.20142	2.10E-06	22.4985418962309	0.0195408	0.0332822	0.159765	0.83497909
OM→DC											
rs148799833	G	T	0.00780919	1.11578	0.231025	1.37E-06	23.3258099110255	0.0161915	-0.132725	0.177493	0.454593268
rs76888680	C	G	0.016129	-2.20673	0.445118	7.14E-07	24.5779484676666	0.030917	-0.00506487	0.134588	0.969980715
rs77438198	T	A	0.0171671	0.847451	0.169682	5.90E-07	24.9433734455661	0.015207	-0.388527	0.181583	0.0323824720985
rs7842524	G	A	0.522881	0.277053	0.0592103	2.88E-06	21.8941826153716	0.597416	-0.00310409	0.045162	0.945202801

Supplementary Material 8: Heterogeneity and pleiotropy tests in the Mendelian random -ization process

Table 1:Heterogeneity in initial two-sample forward Mendelian randomization analysis.

**Exposure**	**Outcome**	**IVW**		**MR egger**		**SNP threshold**
		Cochran's Q	Q-P value	Cochran's Q	Q-P value	
IE	OM	9.712	0.641	9.594	0.567	5×10^{-6}
IE	VOM	15.844	0.198	15.783	0.149	5×10^{-6}
IE	DC	8.880	0.713	7.985	0.715	5×10^{-6}

IE:infective endocarditis;OM:osteomyelitis;VOM:Vertebral Osteomyelitis;DC:Discitis

Table 2:Heterogeneity in initial two-sample reverse Mendelian randomization analysis.

**Exposure**	**Outcome**	**IVW**		**MR egger**		**SNP threshold**
		Cochran's Q	Q-P value	Cochran's Q	Q-P value	
OM	IE	5.730	0.767	5.681	0.683	5×10^{-6}
DC	IE	4.572	0.206	4.506	0.105	5×10^{-6}

IE:infective endocarditis;OM:osteomyelitis;VOM:Vertebral Osteomyelitis;DC:Discitis

Table 3: Pleiotropy in the initial two-sample forward Mendelian randomization analysis.

**Exposure**	**Outcome**	**MR-PRESSO Global test-p**	**MR egger—intercept**	**Intercept -P value**	**SNP threshold**
IE	OM	0.697	-0.011	0.737	5×10^{-6}
IE	VOM	0.218	0.017	15.783	5×10^{-6}
IE	DC	0.728	-0.145	0.364	5×10^{-6}

IE:infective endocarditis;OM:osteomyelitis;VOM:Vertebral Osteomyelitis;DC:Discitis

Table 4: Pleiotropy in the initial two-sample reverse Mendelian randomization analysis.

Exposure	Outcome	MR-PRESSO Global test-p	MR egger—intercept	Intercept -P value	SNP threshold
OM	IE	0.805	0.014	0.831	5×10^{-6}
DC	IE	0.390	-0.014	0.880	5×10^{-6}

IE:infective endocarditis;OM:osteomyelitis;DC:Discitis

Table 5:Heterogeneity in the second two-sample Mendelian randomization analysis.

Exposure	Outcome	IVW		MR egger		SNP threshold
		Cochran's Q	Q-P value	Cochran's Q	Q-P value	
IE	OM	4.571	0.470	2.304	0.680	5×10^{-6}
OM	IE	6.511	0.164	4.374	0.224	4×10^{-6}

IE:infective endocarditis；OM:osteomyelitis

Table 6: Pleiotropy in the second two-sample Mendelian randomization analysis.

Exposure	Outcome	MR-PRESSO Global test-p	MR egger—intercept	Intercept -P value	SNP threshold
IE	OM	0.519	0.036	0.207	5×10^{-6}
OM	IE	0.266	-0.073	0.313	4×10^{-6}

IE:infective endocarditis；OM:osteomyelitis

Supplementary Material 9: A secondary analysis on two-sample Mendelian randomization

Forward two-sample Mendelian randomization

SNP	Effect Allele	Other Allele	Associated with IE					Association with OM			
			EAF	Beta	S.E	P value	F-statistic	EAF	Beta	S.E	P value
OM→IE											
rs113069681	C	T	0.0380086	-0.575655	0.121817	2.29E-06	22.3309118048577	0.0302945	0.0212928	0.0618818	0.730780584
rs118177040	T	C	0.0210943	0.5497	0.118912	3.79E-06	21.3696692479337	0.0222573	-0.123981	0.0771329	0.10797445
rs541727214	T	C	0.0135436	-1.38791	0.257747	7.25E-08	28.9956644539767	0.0101929	0.0711896	0.113854	0.531794694
rs704084	C	T	0.103289	-0.330233	0.0714944	3.86E-06	21.3350950450093	0.0821771	-0.0267122	0.0397895	0.502006106
rs723459	T	C	0.398596	0.188076	0.0399063	2.44E-06	22.211686075195	0.401016	0.0179152	0.0216534	0.408032729
rs9928937	T	G	0.386995	-0.189183	0.041274	4.57E-06	21.0091649179103	0.391525	-0.0133786	0.0217665	0.538790587

Reverse two-sample Mendelian randomization

SNP	Effect Allele	Other Allele	Associated with OM					Association with IE			
			EAF	Beta	S.E	P value	F-statistic	EAF	Beta	S.E	P value
IE→OM											
rs117366634	A	G	0.0121304	-0.465874	0.0952783	1.01E-06	23.9082155320164	0.0371489	0.0282683	0.106305	0.790304
rs12600041	G	A	0.764148	0.115761	0.0249804	3.59E-06	21.474545334547	0.727131	-0.0104759	0.0450005	0.81592
rs12959355	T	C	0.317796	0.108071	0.0227243	1.98E-06	22.6170275251818	0.259764	-0.132895	0.0460076	0.00387035
rs13315366	T	C	0.209699	0.126375	0.0259479	1.11E-06	23.7200784290654	0.201235	-0.019637	0.0496969	0.692743
rs74405814	T	C	0.0200134	0.359722	0.0774363	3.39E-06	21.5795818925962	0.00485702	0.0702928	0.288135	0.807263

Supplementary Material 10: Results of multivariate Mendelian randomization

exposure	outcome	or	or_lci95	or_uci95	pval
diabetes	osteomyelitis	57.898	0.374	8958.310	0.115
endocarditis	osteomyelitis	0.920	0.643	1.317	0.648
obesity	osteomyelitis	4936180704.364	$5.631e^{-11}$	$4.327e^{+29}$	0.341
smoking	osteomyelitis	2.886	0.078	107.122	0.565

Detailed information on multivariate Mendelian randomized SNPs

SNP	Exposure	Effect Allele	Other Allele	EAF	Beta	S.E	P value
rs10195252	smoke	C	T	0.405073	-0.000699671	0.00115948	0.550000393
rs11096777	smoke	C	T	0.18038	0.00945455	0.00148135	1.70000422216e-10
rs11210229	smoke	G	A	0.616306	-0.00599733	0.00116916	2.90001336905e-07
rs112674299	smoke	T	C	0.133398	0.00187083	0.00167431	0.259999792
rs1149331	smoke	A	G	0.52345	0.0057394	0.00113701	4.49997395326e-07
rs11688682	smoke	C	G	0.270982	-0.00284303	0.00132362	0.0320000015974
rs11712037	smoke	G	C	0.118751	-0.00182736	0.00175883	0.299999824
rs11720108	smoke	T	C	0.246322	-0.00124397	0.00132155	0.349999964
rs12140153	smoke	T	G	0.094229	-0.00391491	0.00199679	0.0499999995008
rs12469094	smoke	C	A	0.68186	-0.00685575	0.00122457	2.19998641984e-08
rs1260326	smoke	C	T	0.60428	-0.00162433	0.00116209	0.160000006
rs12693975	smoke	A	G	0.813989	0.0079645	0.00146069	4.99999995008e-08
rs12739866	smoke	T	C	0.101647	0.00189618	0.00203779	0.349999964
rs13414381	smoke	C	T	0.127855	-0.000152525	0.00170788	0.929999896
rs1429423	smoke	T	C	0.783461	-0.0072752	0.00138409	1.49999565138e-07
rs1496653	smoke	G	A	0.20345	0.00308337	0.00141221	0.0290001336905
rs2476601	smoke	G	A	0.899231	0.00393848	0.00188591	0.0369998531173
rs2494195	smoke	C	T	0.595344	-4.64E-05	0.0011587	0.969999923
rs2793829	smoke	T	C	0.107449	-0.000712706	0.00183808	0.699999936
rs28545614	smoke	T	C	0.156729	0.00893084	0.00157156	1.29998996538e-08
rs2997447	smoke	A	G	0.210951	-0.00105572	0.00140781	0.450000504
rs3001723	smoke	A	G	0.301175	0.00721497	0.00123869	5.69993627094e-09
rs34563765	smoke	C	A	0.157554	0.00111352	0.00156264	0.479999738
rs348330	smoke	A	G	0.632156	0.0018919	0.00118411	0.11000008
rs3773814	smoke	C	A	0.152382	0.00792956	0.00158658	5.80002679602e-07
rs4660303	smoke	A	G	0.225108	0.00158655	0.00136248	0.239999866
rs4686471	smoke	C	T	0.610075	-0.000625009	0.00117185	0.589999984
rs4689394	smoke	G	C	0.600917	0.00171925	0.00116398	0.139999988

rs4832280	smoke	T	C	0.394969	0.00629718	0.00116336	6.19997588072e-08
rs58432198	smoke	T	C	0.11736	-0.0030035	0.00178143	0.0920004602516
rs62286986	smoke	C	G	0.183814	0.000597406	0.00147697	0.689999856
rs6727997	smoke	G	A	0.654335	0.00737776	0.00120444	8.99994799637e-10
rs7563362	smoke	G	A	0.856134	0.0057207	0.00162807	0.00043999728836
rs7569203	smoke	C	A	0.311269	0.00691663	0.00123291	2.00000001997e-08
rs7615045	smoke	G	A	0.315803	-0.000434884	0.00122436	0.719999177
rs9848655	smoke	A	G	0.159923	-0.00235267	0.00155437	0.129999895
rs10195252	diabetes	C	T	0.405107	-0.00353224	6.29941180549e-15	6.29941180549e-15
rs11096777	diabetes	C	T	0.180354	-0.000329524	0.570000189	0.570000189
rs11210229	diabetes	G	A	0.616344	0.000376723	0.410000135	0.410000135
rs112674299	diabetes	T	C	0.133392	-0.00372576	1.19999655705e-08	1.19999655705e-08
rs1149331	diabetes	A	G	0.523442	0.000654356	0.139999988	0.139999988
rs11688682	diabetes	C	G	0.270977	-0.00274655	1.09999319894e-07	1.09999319894e-07
rs11712037	diabetes	G	C	0.118748	-0.00453668	4.00036851046e-11	4.00036851046e-11
rs11720108	diabetes	T	C	0.246346	-0.00408117	2.70022528689e-15	2.70022528689e-15
rs12140153	diabetes	T	G	0.09422	-0.00384642	8.1999272627e-07	8.1999272627e-07
rs12469094	diabetes	C	A	0.681832	-1.92E-05	0.969999923	0.969999923
rs1260326	diabetes	C	T	0.604269	0.0036418	1e-15	1e-15
rs12693975	diabetes	A	G	0.813968	0.00110696	0.0519995996534	0.0519995996534
rs12739866	diabetes	T	C	0.101628	0.00410896	2.49999995008e-07	2.49999995008e-07
rs13414381	diabetes	C	T	0.127892	-0.00536851	8.4996295945e-16	8.4996295945e-16
rs1429423	diabetes	T	C	0.783467	-0.000123895	0.82000009	0.82000009
rs1496653	diabetes	G	A	0.203507	-0.00395496	7.50066898736e-13	7.50066898736e-13
rs2476601	diabetes	G	A	0.899214	-0.00437024	2.99999133272e-09	2.99999133272e-09
rs2494195	diabetes	C	T	0.59536	0.00236592	1.70000422216e-07	1.70000422216e-07
rs2793829	diabetes	T	C	0.107403	0.00447507	4.60002296665e-10	4.60002296665e-10
rs28545614	diabetes	T	C	0.156728	0.00227508	0.000210000341027	0.000210000341027
rs2997447	diabetes	A	G	0.210977	-0.00279399	3.79996853037e-07	3.79996853037e-07
rs3001723	diabetes	A	G	0.301188	-0.000210229	0.660000098	0.660000098
rs34563765	diabetes	C	A	0.157562	-0.00109028	0.0739997069734	0.0739997069734
rs348330	diabetes	A	G	0.632179	-0.00245959	1.09999319894e-07	1.09999319894e-07
rs3773814	diabetes	C	A	0.152386	-3.10E-05	0.959999927	0.959999927
rs4660303	diabetes	A	G	0.225102	0.00352585	3.50025754158e-11	3.50025754158e-11
rs4686471	diabetes	C	T	0.610018	0.00277132	1.40000633224e-09	1.40000633224e-09
rs4689394	diabetes	G	C	0.600901	0.00436989	7.19946127441e-22	7.19946127441e-22
rs4832280	diabetes	T	C	0.394972	-0.000330924	0.470000154	0.470000154
rs58432198	diabetes	T	C	0.117393	-0.00394295	1.49999565138e-08	1.49999565138e-08
rs62286986	diabetes	C	G	0.18382	0.00308942	8.60003066889e-08	8.60003066889e-08

rs6727997	diabetes	G	A	0.654333	-0.000796667	0.0899994799637	0.0899994799637
rs7563362	diabetes	G	A	0.856178	0.00334506	1.49999565138e-07	1.49999565138e-07
rs7569203	diabetes	C	A	0.311297	0.000248192	0.610000232	0.610000232
rs7615045	diabetes	G	A	0.315753	0.00538022	2.29985258669e-29	2.29985258669e-29
rs9848655	diabetes	A	G	0.159943	0.00307593	4.09996359042e-07	4.09996359042e-07
rs10195252	endocarditis	C	T	0.405066	-0.0225606	0.616247258	0.616247258
rs11096777	endocarditis	C	T	0.180362	-0.0516708	0.369342258	0.369342258
rs11210229	endocarditis	G	A	0.616292	-0.0354404	0.434340173	0.434340173
rs112674299	endocarditis	T	C	0.133385	-0.0219233	0.73510267	0.73510267
rs1149331	endocarditis	A	G	0.523486	-0.0801488	0.0694960306431	0.0694960306431
rs11688682	endocarditis	C	G	0.270992	-0.00647493	0.899279721	0.899279721
rs11712037	endocarditis	G	C	0.118744	-0.0799417	0.241295376	0.241295376
rs11720108	endocarditis	T	C	0.24632	-0.0418879	0.413381465	0.413381465
rs12140153	endocarditis	T	G	0.0942022	-0.111469	0.14976592	0.14976592
rs12469094	endocarditis	C	A	0.681859	-0.0350128	0.46111792	0.46111792
rs1260326	endocarditis	C	T	0.604295	0.022624	0.615440398	0.615440398
rs12693975	endocarditis	A	G	0.813998	0.116042	0.0400894409476	0.0400894409476
rs12739866	endocarditis	T	C	0.10165	0.0541642	0.492148312	0.492148312
rs13414381	endocarditis	C	T	0.127853	0.14311	0.0302872609934	0.0302872609934
rs1429423	endocarditis	T	C	0.783446	0.042717	0.427316832	0.427316832
rs1496653	endocarditis	G	A	0.203448	0.0229728	0.674639864	0.674639864
rs2476601	endocarditis	G	A	0.899223	-0.0200832	0.783054519	0.783054519
rs2494195	endocarditis	C	T	0.595349	0.0941357	0.0359211308137	0.0359211308137
rs2793829	endocarditis	T	C	0.107408	0.0204476	0.775187927	0.775187927
rs28545614	endocarditis	T	C	0.156706	0.0652341	0.285413821	0.285413821
rs2997447	endocarditis	A	G	0.210948	0.00904183	0.868146063	0.868146063
rs3001723	endocarditis	A	G	0.301212	0.0241508	0.615406388	0.615406388
rs34563765	endocarditis	C	A	0.157577	-0.0507769	0.400698761	0.400698761
rs348330	endocarditis	A	G	0.632173	0.0490822	0.284633491	0.284633491
rs3773814	endocarditis	C	A	0.152363	-0.0485486	0.431455491	0.431455491
rs4660303	endocarditis	A	G	0.225115	-0.0283693	0.591252514	0.591252514
rs4686471	endocarditis	C	T	0.610104	-0.0374135	0.409521776	0.409521776
rs4689394	endocarditis	G	C	0.600887	-0.0459443	0.307934962	0.307934962
rs4832280	endocarditis	T	C	0.394991	-0.0449915	0.318583295	0.318583295
rs58432198	endocarditis	T	C	0.117355	0.108402	0.114189455	0.114189455
rs62286986	endocarditis	C	G	0.183792	-0.0433662	0.45012797	0.45012797
rs6727997	endocarditis	G	A	0.6543	0.0218032	0.640352339	0.640352339
rs7563362	endocarditis	G	A	0.856149	0.0518705	0.4114452	0.4114452
rs7569203	endocarditis	C	A	0.311242	0.0151854	0.750408941	0.750408941

rs7615045	endocarditis	G	A	0.315787	0.0601663	0.20528537	0.20528537
rs9848655	endocarditis	A	G	0.159923	-0.119663	0.0465211026618	0.0465211026618
rs10195252	Obesity	C	T	0.40506	0.000281218	0.000212136	0.180000205
rs11096777	Obesity	C	T	0.18036	-0.000329221	0.000270985	0.220000162
rs11210229	Obesity	G	A	0.616287	2.25661e-05	0.000213914	0.919999942
rs112674299	Obesity	T	C	0.133382	-0.00056519	0.000306281	0.0649994976198
rs1149331	Obesity	A	G	0.523474	0.000379964	0.000208028	0.0680001702441
rs11688682	Obesity	C	G	0.271002	-2.57E-05	0.000242155	0.919999942
rs11712037	Obesity	G	C	0.118745	0.000698151	0.000321719	0.0299999133272
rs11720108	Obesity	T	C	0.246312	-0.000370787	0.000241732	0.129999895
rs12140153	Obesity	T	G	0.094205	-0.00114382	0.000365377	0.00170000422216
rs12469094	Obesity	C	A	0.681847	-0.000102552	0.000224039	0.649999466
rs1260326	Obesity	C	T	0.604293	0.000328476	0.000212615	0.119999932
rs12693975	Obesity	A	G	0.813997	0.000380582	0.00026724	0.149999911
rs12739866	Obesity	T	C	0.101649	0.00034638	0.000372817	0.349999964
rs13414381	Obesity	C	T	0.127852	0.000484826	0.000312471	0.119999932
rs1429423	Obesity	T	C	0.783453	0.000359171	0.000253227	0.160000006
rs1496653	Obesity	G	A	0.203441	6.81352e-05	0.000258333	0.789999834
rs2476601	Obesity	G	A	0.899224	-0.000641089	0.000345041	0.0629999202947
rs2494195	Obesity	C	T	0.595342	-0.000351471	0.000211999	0.0969996126511
rs2793829	Obesity	T	C	0.107417	-0.000164939	0.000336351	0.620000443
rs28545614	Obesity	T	C	0.156707	0.000354825	0.000287537	0.220000162
rs2997447	Obesity	A	G	0.210948	-0.000212288	0.000257575	0.410000135
rs3001723	Obesity	A	G	0.301204	0.000432777	0.000226624	0.0560002544078
rs34563765	Obesity	C	A	0.157579	-0.00146683	0.000285881	2.90001336905e-07
rs348330	Obesity	A	G	0.632172	0.000205748	0.00021665	0.340000065
rs3773814	Obesity	C	A	0.15237	9.95014e-05	0.000290213	0.730000235
rs4660303	Obesity	A	G	0.225111	0.000601717	0.000249284	0.016000001
rs4686471	Obesity	C	T	0.610091	0.000343226	0.000214351	0.11000008
rs4689394	Obesity	G	C	0.600884	-0.000218666	0.000212909	0.299999824
rs4832280	Obesity	T	C	0.394985	-0.000185036	0.000212847	0.380000353
rs58432198	Obesity	T	C	0.117351	-9.18E-05	0.00032594	0.780000714
rs62286986	Obesity	C	G	0.183791	-2.79E-05	0.000270187	0.919999942
rs6727997	Obesity	G	A	0.65431	-8.71E-06	0.000220355	0.969999923
rs7563362	Obesity	G	A	0.85615	0.000828142	0.000297872	0.00539995325072
rs7569203	Obesity	C	A	0.311246	-0.000151854	0.000225565	0.499999995
rs7615045	Obesity	G	A	0.315792	-0.000375171	0.000223968	0.0940004645745
rs9848655	Obesity	A	G	0.159914	-0.000240303	0.000284322	0.400000008

Supplementary Material 11: Sensitivity analysis of two-sample Mendelian randomization

A

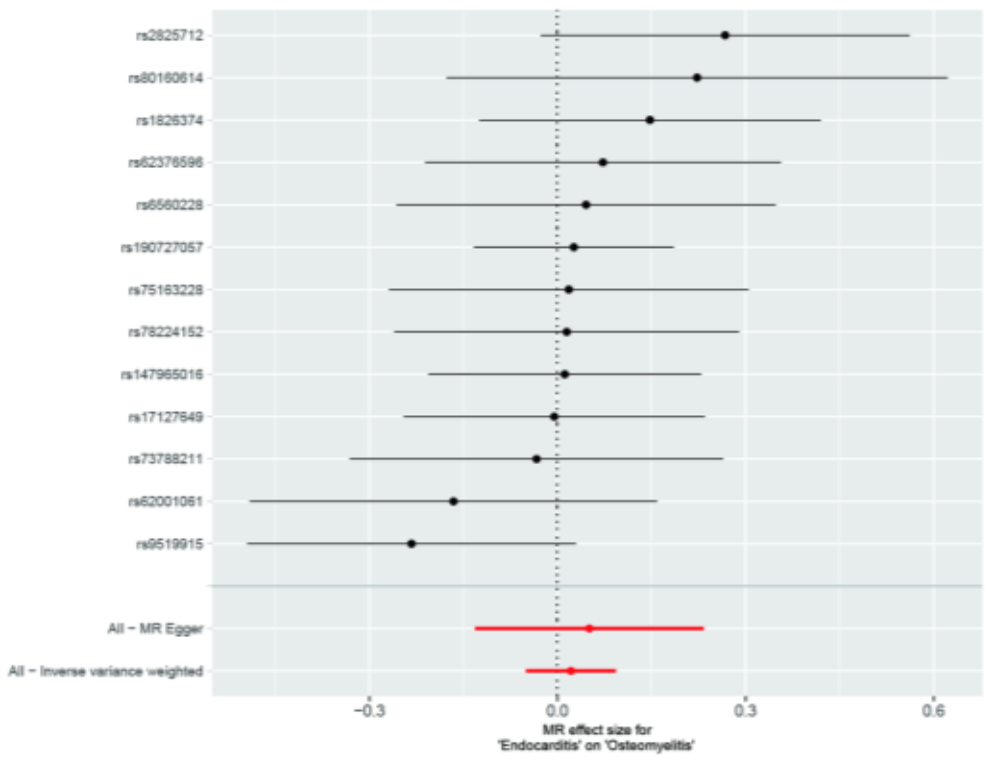

B

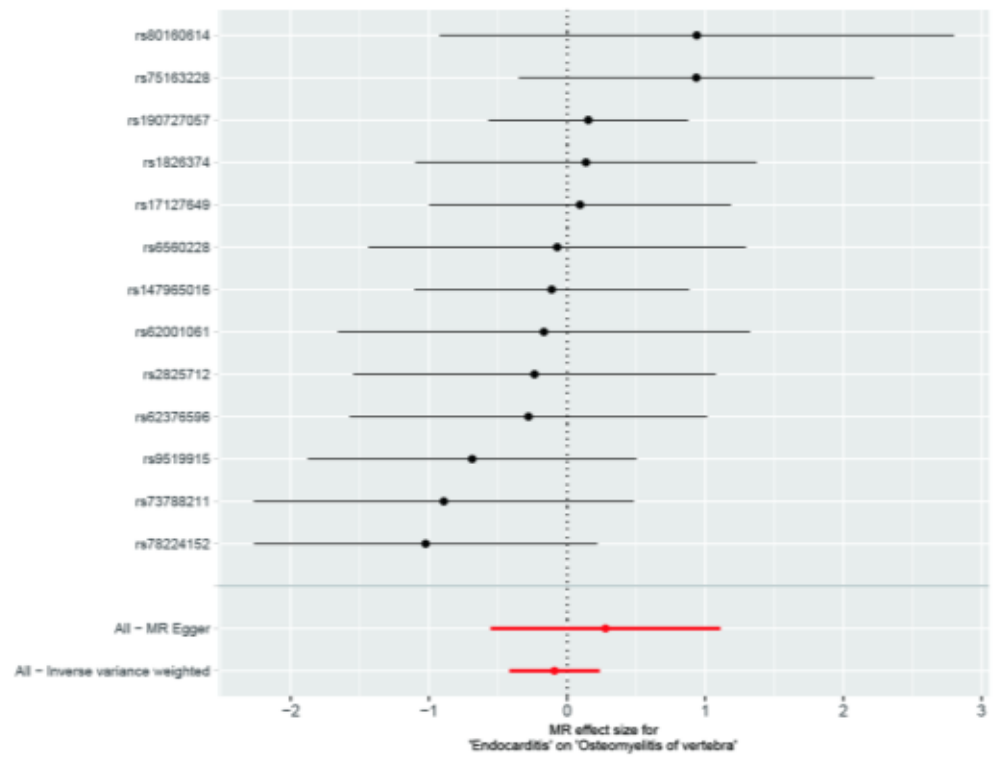

C

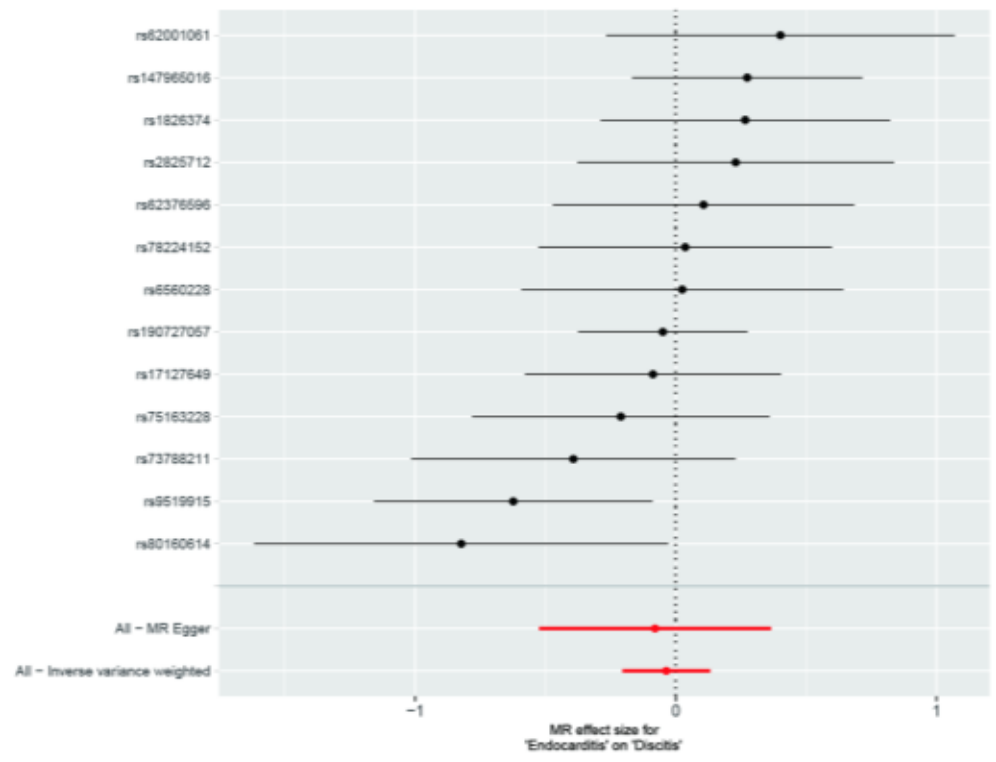

D

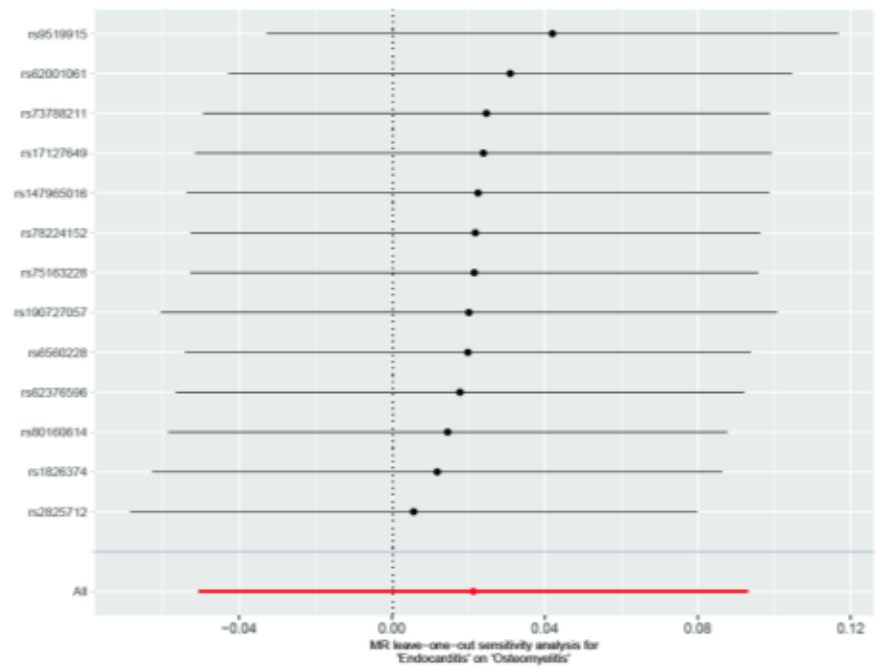

E

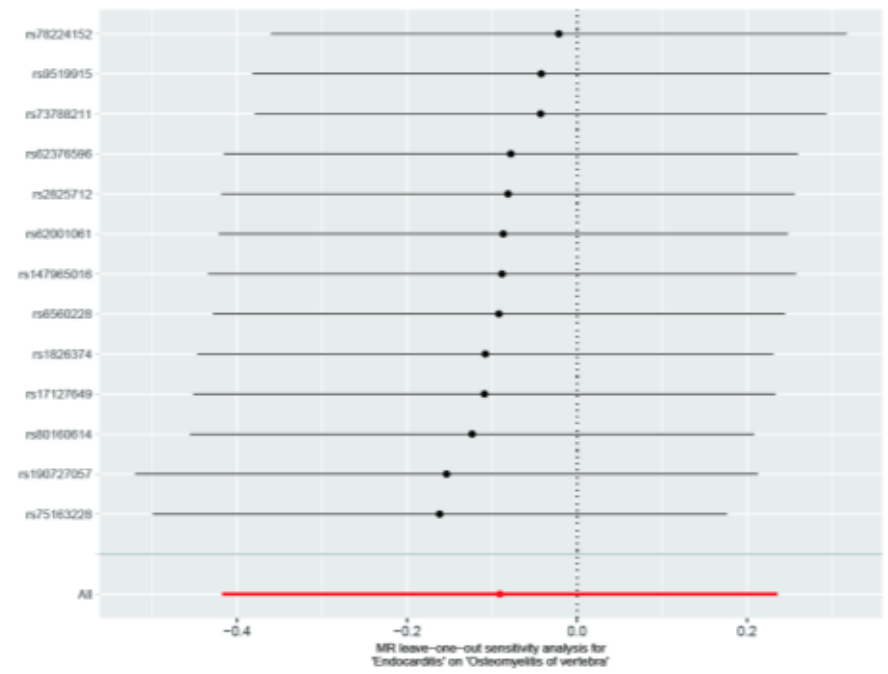

F

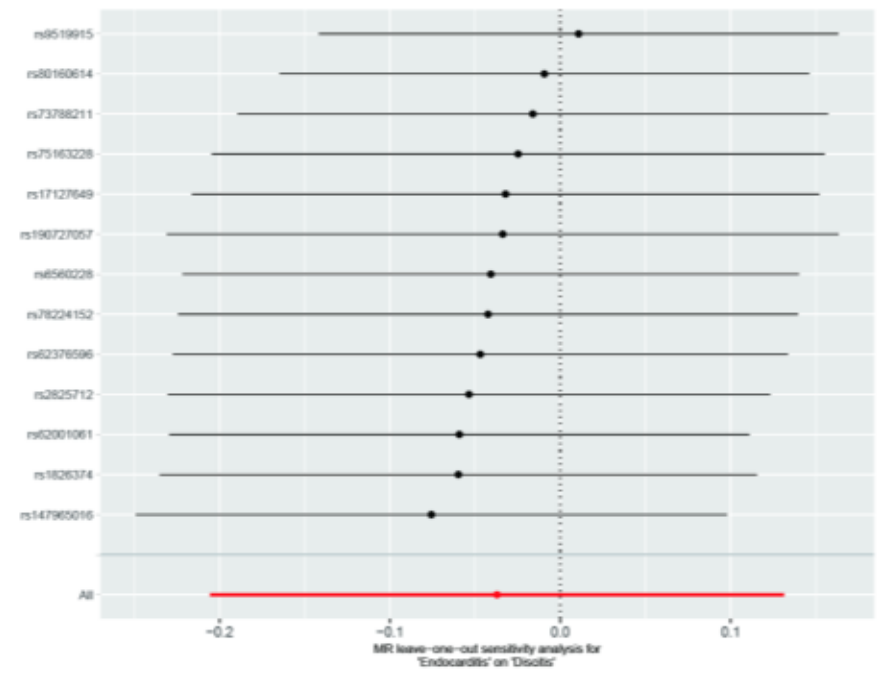

Initial two-sample Mendelian randomization

A:Forest plot of endocarditis as exposure and osteomyelitis as outcome;B:Forest plot of endocarditis as exposure and vertebral osteomyelitis as outcome;C:

Forest plot of endocarditis as exposure and discitis as outcome;D:Sensitivity analysis of endocarditis as exposure and osteomyelitis as outcome;E:Sensitivity analysis of endocarditis as exposure and vertebral osteomyelitis as outcome;F:Sensitivity analysis of endocarditis as exposure and discitis as outcome.

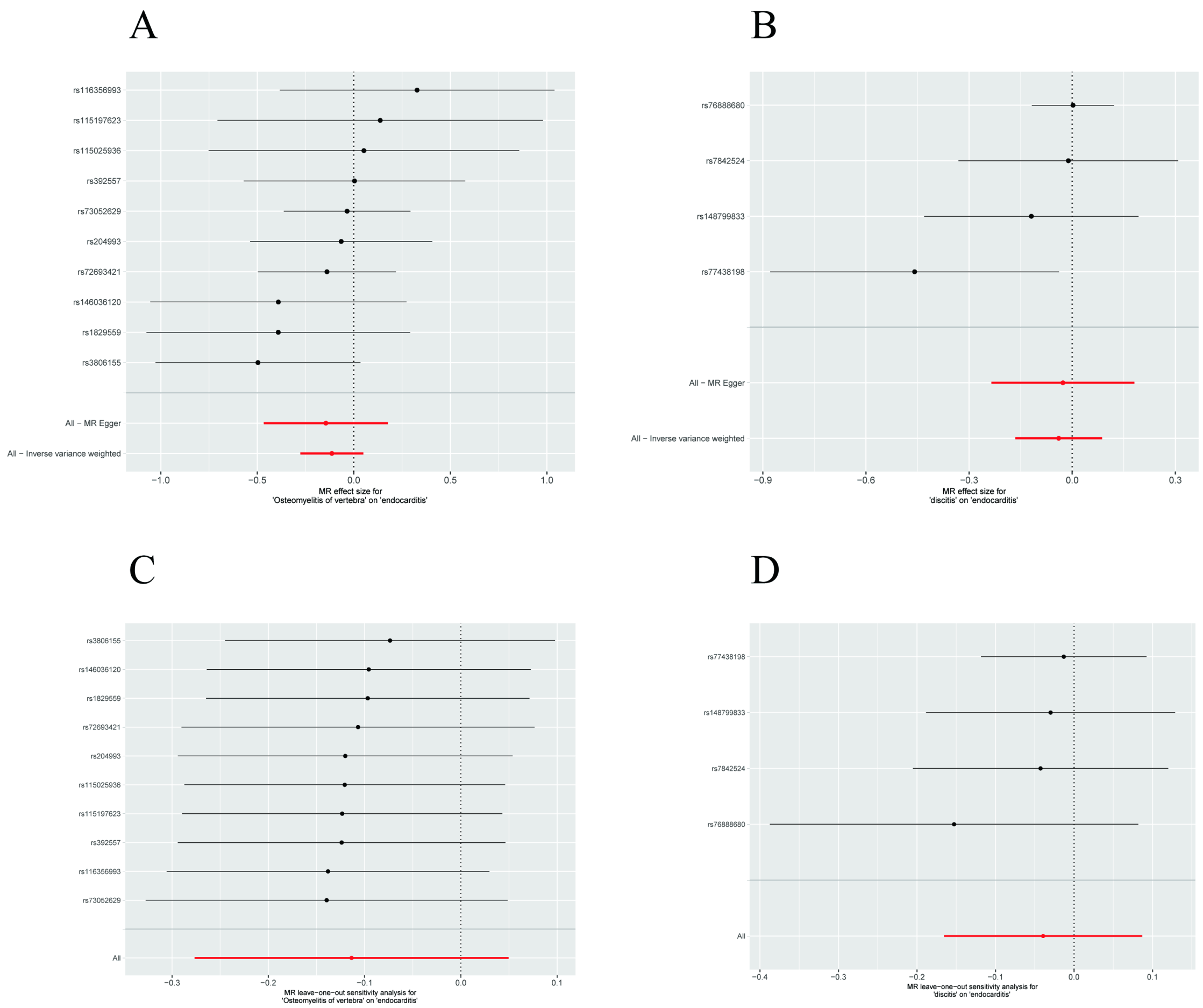

Initial two-sample Mendelian randomization

A:Osteomyelitis as an exposure and endocarditis as an ending forest plot;B:Forest plot of discitis as exposure and endocarditis as outcome;C:Sensitivity analysis of osteomyelitis as exposure and endocarditis as outcome;D:Sensitivity analysis of discitis as exposure and endocarditis as outcome.

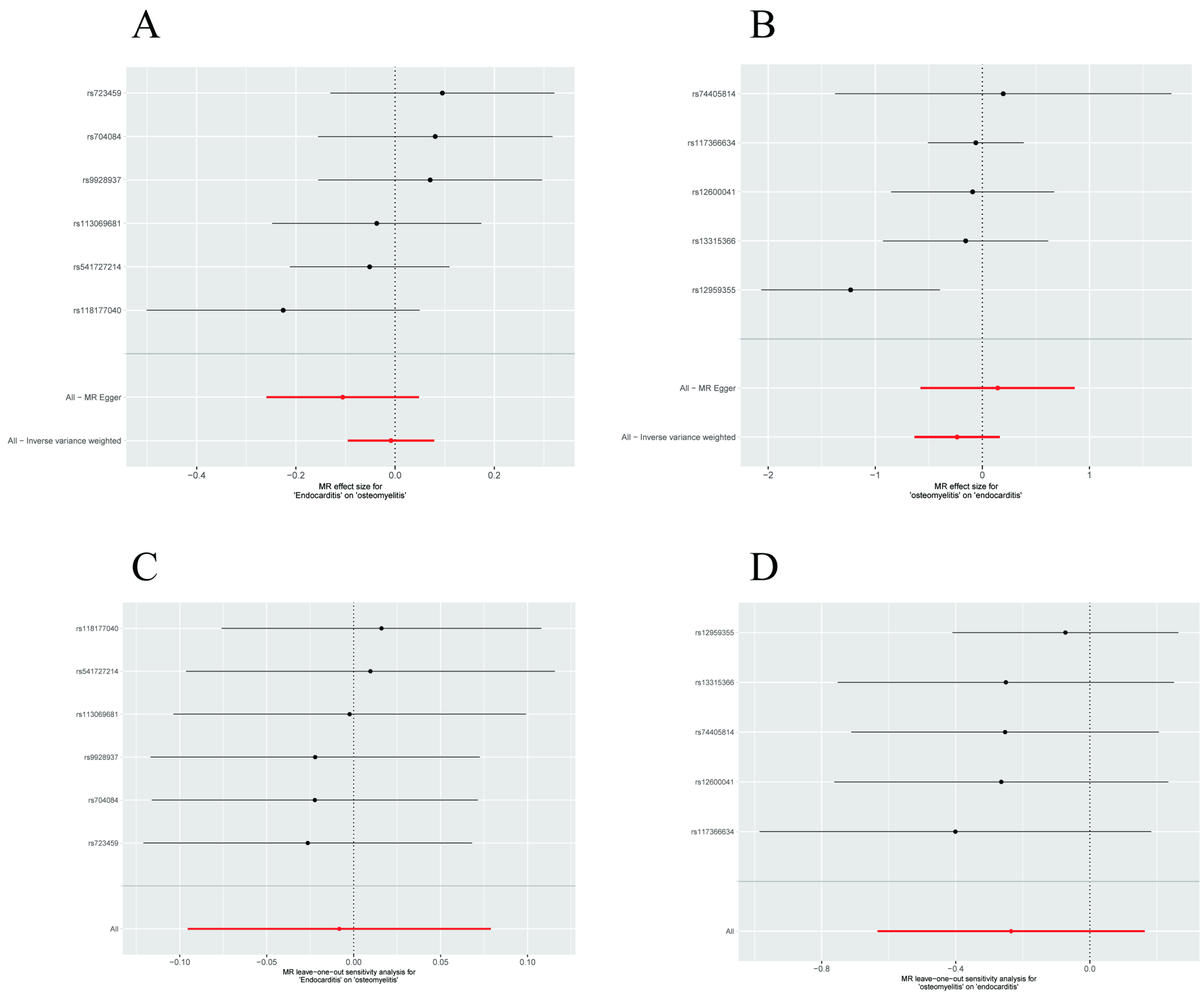

Second two-sample Mendelian randomization

A:Forest plot of endocarditis as exposure and osteomyelitis as outcome;B:Osteomyelitis as an exposure and endocarditis as an ending forest plot;C:Sensitivity analysis of endocarditis as exposure and osteomyelitis as outcome;D:Sensitivity analysis of osteomyelitis as exposure and endocarditis as outcome

Supplementary Material 12: Combined results of Mata analysis of IE and OM two-sample Mendelian randomized IVW results

Meta-analysis of forward Mendelian randomization

Study	logOR	SE(logOR)	OR	95%-CI	Weight (common)	Weight (random)
A	0.0218	0.0367	1.02	[0.95; 1.10]	59.5%	59.5%
B	−0.0080	0.0444	0.99	[0.91; 1.08]	40.5%	40.5%
**Common effect model**			**1.01**	**[0.96; 1.07]**	**100.0%**	**.**
**Random effects model**			**1.01**	**[0.96; 1.07]**	**.**	**100.0%**

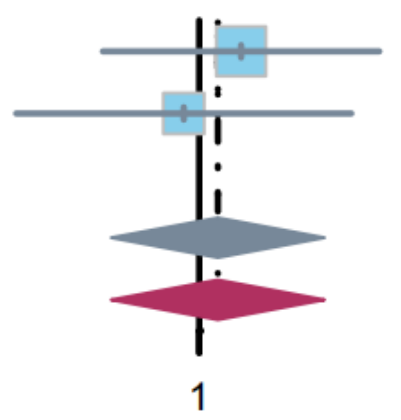

Heterogeneity: $I^2 = 0.0\%$, $\tau^2 = 0$, $p = 0.6051$

A:Initial analysis;B:Secondary analysis

Meta-analysis of reverse Mendelian randomization

Study	logOR	SE(logOR)	OR	95%-CI	Weight (common)	Weight (random)
A	−0.1143	0.0834	0.89	[0.76; 1.05]	78.6%	78.6%
B	−0.2345	0.1597	0.79	[0.58; 1.08]	21.4%	21.4%
**Common effect model**			**0.87**	**[0.75; 1.00]**	**100.0%**	**.**
**Random effects model**			**0.87**	**[0.75; 1.00]**	**.**	**100.0%**

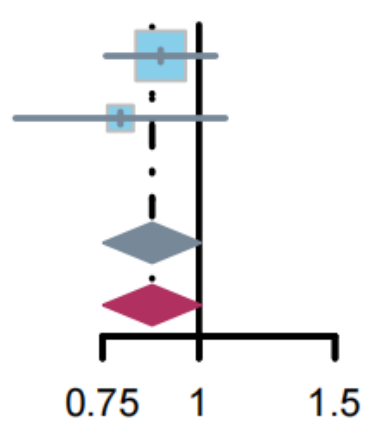

Heterogeneity: $I^2 = 0.0\%$, $\tau^2 = 0$, $p = 0.5048$

A:Initial analysis;B:Secondary analysis
